# Lobosteroids A–F: Six New Highly Oxidized Steroids from the Chinese Soft Coral *Lobophytum* sp.

**DOI:** 10.3390/md21080457

**Published:** 2023-08-19

**Authors:** Zi-Yi Xia, Man-Man Sun, Yang Jin, Li-Gong Yao, Ming-Zhi Su, Lin-Fu Liang, Hong Wang, Yue-Wei Guo

**Affiliations:** 1 Collaborative Innovation Center of Yangtze River Delta Region Green Pharmaceuticals and College of Pharmaceutical Science, Zhejiang University of Technology, Hangzhou 310014, China; xiaziyi@simm.ac.cn; 2 Shandong Laboratory of Yantai Drug Discovery, Bohai Rim Advanced Research Institute for Drug Discovery, 198 Binhai East Road, High-Tech Zone, Yantai 264117, China; mmsun@baridd.ac.cn (M.-M.S.); yang_jin98@163.com (Y.J.); yaoligong@simm.ac.cn (L.-G.Y.); smz0310@163.com (M.-Z.S.); 3 School of Medicine, Shanghai University, 99 Shangda Road, Bao Shan District, Shanghai 200444, China; 4 College of Materials Science and Engineering, Central South University of Forestry and Technology, 498 South Shaoshan Road, Changsha 410004, China

**Keywords:** soft coral, *Lobophytum* sp., steroids, antibacterial activity

## Abstract

To explore the steroidal constituents of the soft coral *Lobophytum* sp. at the coast of Xuwen County, Guangdong Province, China, a chemical investigation of the above-mentioned soft coral was carried out. After repeated column chromatography over silica gel, Sephadex LH-20, and reversed-phase HPLC, six new steroids, namely lobosteroids A–F (**1**–**6**), along with four known compounds **7**–**10**, were obtained. Their structures were determined by extensive spectroscopic analysis and comparison with the spectral data reported in the literature. Among them, the absolute configuration of **1** was determined by X-ray diffraction analysis using Cu K*α* radiation. These steroids were characterized by either the presence of an *α*,*β*-*α*′,*β*′-unsaturated carbonyl, or an *α*,*β*-unsaturated carbonyl moiety in ring A, or the existence of a 5*α*,8*α*-epidioxy system in ring B, as well as diverse oxidation of side chains. The antibacterial bioassays showed that all isolated steroids exhibited significant inhibitory activities against the fish pathogenic bacteria *Streptococcus parauberis* FP KSP28, *Phoyobacterium damselae* FP2244, and *Streptococcus parauberis* SPOF3K, with IC_90_ values ranging from 0.1 to 11.0 µM. Meanwhile, compounds **2** and **6**–**10** displayed potent inhibitory effects against the vancomycin-resistant *Enterococcus faecium* bacterium G7 with IC_90_ values ranging from 4.4 to 18.3 µM. Therefore, ten highly oxidized steroids with strong antibacterial activities were isolated from the Chinese soft coral *Lobophytum* sp., which could be developed as new chemotypes of antibacterial drug leads.

## 1. Introduction

Unlike terrestrial, the unique and complex marine environment creates rich chemodiversities and biodiversities of secondary metabolites in soft corals [1], such as the bioactive steroids with diverse structural features [2]. Structurally, these soft coral-derived steroids display an array of carbon frameworks, ranging from the usual cholestane [3], ergostane [4], and pregnane-type [5] sterols to the rare secosteroids [6] and highly degraded steroids [7]. Biologically, this group of secondary metabolites exhibit a wide spectrum of bioactivities, including antibacterial [8], anti-inflammatory [9], cytotoxic [10], immunosuppressive [11], and PTP1B inhibitory [6] activities. These properties make steroids attract the continuous attention of chemists and pharmacologists [12].

It is well known that soft corals of the genus *Lobophytum* are one group of the most important marine invertebrates widely distributed in waters. They produce a wealthy biochemical repository of secondary metabolites [13] ranging from terpenoids [14], steroids [15], prostaglandins [16], and amides [17] to quinones [18]. Among these chemical constituents, steroids are one major group of metabolites that were found in many species of the genus *Lobophytum*, including *Lobophytum sarcophytoides* [9,17], *Lobophytum pauciflorum* [15], *Lobophytum michaelae* [19], *Lobophytum crassum* [20,21], *Lobophytum lobophytum* [22], *Lobophytum compactum* [23], *Lobophytum patulum* [24], and *Lobophytum* spp. [10,25,26,27]. Notably, these metabolites display various biological activities, such as anticancer [10,15,25], anti-inflammatory [9,17,19], and 5*α*-reductase inhibitory [28] activities.

**Figure 1:**
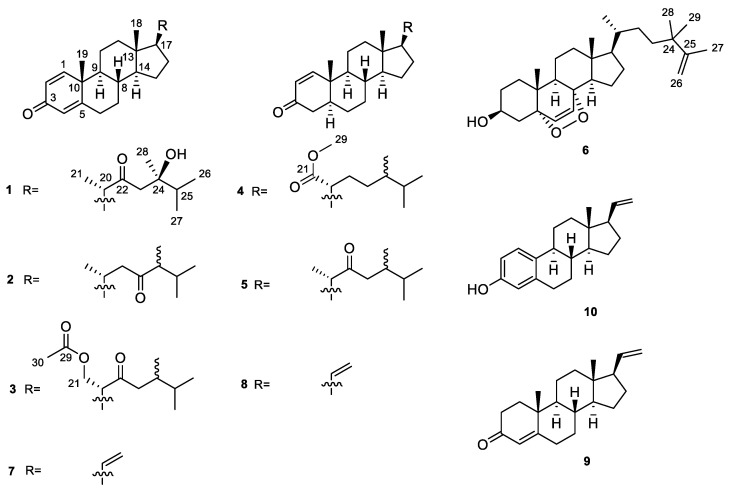
Chemical structures of compounds **1**–**10**.

As part of our ongoing research aimed at discovering bioactive substances from marine invertebrates in China [29], we recently collected *Lobophytum* sp. at the coast of Xuwen County, Guangdong Province, China. In our recent study, we have reported the isolation and structural elucidation of anti-tumor cembrane diterpenoids from the Hainan specimens of *Lobophytum* sp. [30]. While our current investigation on the Guangdong collection of *Lobophytum* sp. has now resulted in the isolation of six new steroids, lobosteroids A–F (**1**–**6**), together with four known analogs **7**–**10** (Figure 1). The structural difference of six new steroids **1**–**6** is mainly attributed to the different degrees of oxidation in rings A and B of the steroidal nucleus and the variations of functional groups on the side chains. This paper describes the isolation, structural elucidation, and bioactivity of these compounds.

## 2. Results and Discussion

The frozen animals were cut into pieces and extracted with acetone exhaustively. The Et_2_O-soluble portion of the acetone extract was repeatedly column chromatographed over silica gel, Sephadex LH-20, and reversed-phase HPLC to yield ten pure steroids **1**–**10** (Figure 1). Four known steroids were readily identified as pregna-1,4,20-trien-3-one (**7**) [31], pregna-1,20-dien-3-one (**8**) [32], pregna-4,20-dien-3-one (**9**) [33], and 19-norpregna-1,3,5(10),20-tetraen-3-ol (**10**) [34], respectively, by comparison of their NMR data and optical rotation [α]_D_ values with those reported in the literature.

**Figure 2:**
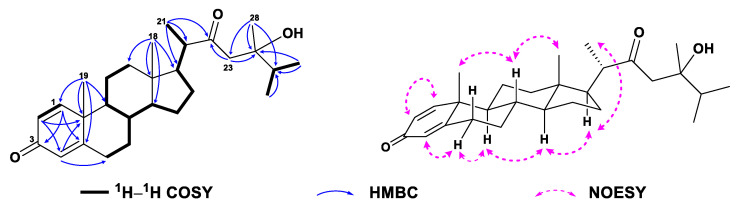
^1^H–^1^H COSY, selected key HMBC and NOE correlations of **1**.

**Figure 3:**
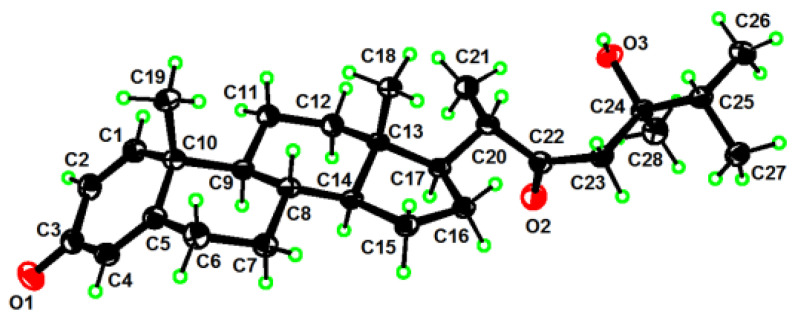
Perspective ORTEP drawing of **1** (displacement ellipsoids are drawn at the 50% probability level).

Compound **1**, colorless crystals, had the molecular formula of C_28_H_42_O_3_ as established by HRESIMS (Figure S7) from the protonated molecular ion peak observed at *m*/*z* 427.3210 [M + H]^+^ (calcd. 427.3207), implying eight degrees of unsaturation. Extensive analysis of ^13^C NMR and DEPT spectra of **1** (Table 1, Figure S2) disclosed the presence of 28 carbons, consisting of six methyls, seven sp^3^ methylenes, six sp^3^ methines, three sp^3^ quaternary carbons (including one oxygenated at *δ*_C_ 74.0), three sp^2^ methines (*δ*_C_ 124.1, 127.7, and 155.8), and three sp^2^ quaternary carbons (including one olefinic at *δ*_C_ 169.1 and two carbonylic at *δ*_C_ 186.5 and 217.6). Thus, compound **1** still required a four-ring system to satisfy the remaining four degrees of unsaturation. Considering the co-isolated known metabolites, compound **1** was likely a steroid whose basic nucleus was a fused four-ring carbon framework. Overall, the gross ^1^H and ^13^C spectral data of **1** (Table 1 and Table 2, Figures S1 and S2) were reminiscent of 24-methylenecholesta-1,4,24(28)-trien-3-one, a sterol previously reported from the soft coral *Dendronephthya studeri* [35]. Careful comparison of their NMR data revealed they possessed the same steroidal nucleus possessing an *α*,*β*-*α*′,*β*′-unsaturated carbonyl moiety, which was straightforward from NMR signals at *δ*_H_ 7.05 (d, *J* = 10.2 Hz, H-1), 6.23 (dd, *J* = 10.2, 2.0 Hz, H-2), 6.07 (br s, H-4), and *δ*_C_ 155.8 (d, C-1), 127.7 (d, C-2), 186.5 (s, C-3), 124.1 (d, C-4), 169.1 (s, C-5) (Figure S3). The establishment of ring A was further confirmed by the key HMBC correlations from H_3_-19 (*δ*_H_ 1.10) to C-1 (*δ*_C_ 155.8), C-5 (*δ*_C_ 169.1), C-9 (*δ*_C_ 52.3), and C-10 (*δ*_C_ 43.6), from H-1 (*δ*_H_ 7.05) to C-3 (*δ*_C_ 186.5) and C-5, from H-2 (*δ*_H_ 6.23) to C-4 (*δ*_C_ 124.1) and C-10, and from H-4 (*δ*_H_ 6.07) to C-6 (*δ*_C_ 33.0) and C-10 (Figure 2 and Figure S4). However, they differed in the structures of their side chains. First, the methylene C-22 in 24-methylenecholesta-1,4,24(28)-trien-3-one was oxidized into a ketone in **1**, which was characterized by the remarkably down-field chemical shift of *δ*_C_ 217.6 (s, C-22). Secondly, the terminal double bond Δ^24(28)^ in 24-methylenecholesta-1,4,24(28)-trien-3-one was reduced in **1**, accompanied with the hydroxylation at C-24, which was indicated by the NMR signals at *δ*_H_ 1.12 (s, H_3_-28) and *δ*_C_ 23.0 (q, C-28), 74.0 (s, C-24). Furthermore, the structure of the side chain in **1** was verified clearly by the HMBC correlations from H_3_-21 (*δ*_H_ 1.10) to C-17 (*δ*_C_ 52.0), C-20 (*δ*_C_ 50.9) and C-22 (*δ*_C_ 217.6), from H_2_-23 (*δ*_H_ 2.53, 2.66) to C-22 and C-24 (*δ*_C_ 74.0), and from H_3_-28 to C-23 (*δ*_C_ 48.2), C-24 and C-25 (*δ*_C_ 37.4) (Figure 2 and Figure S4). Herein, the planar structure of **1** was determined as depicted in Figure 1. The observed NOE correlations regarding the chiral centers C-8, C-9, C-10, C-13, C-14, C-17, and C-20, and the double bonds Δ^1^ and Δ^4^ of **1** (Figure 2 and Figure S6) were similar to those of 24-methylenecholesta-1,4,24(28)-trien-3-one, suggesting they shared the same relative configurations for these stereocenters and double bonds. However, there were insufficient NOE correlations to assign the relative configuration of C-24. Luckily, suitable single crystals of **1** in MeOH were obtained. The X-ray crystallographic analysis using Cu K*α* radiation (*λ* = 1.54178 Å) firmly disclosed the absolute configuration of **1** was 8*S*,9*S*,10*R*,13*S*,14*S*,17*R*,20*S*,24*R* (Flack parameter: 0.09 (8), Figure 3).

**Figure 4:**
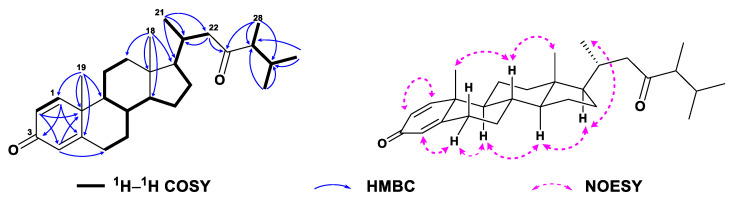
^1^H–^1^H COSY, selected key HMBC and NOE correlations of **2**.

Compound **2** was obtained as a white amorphous powder and it displayed a protonated molecular ion peak at *m*/*z* 411.3255 ([M + H]^+^; calcd. 411.3258) in the HRESIMS spectrum (Figure S15), consistent with a molecular formula of C_28_H_42_O_2_. Inspection of the NMR data of compound **2** (Table 1 and Table 2, Figures S9 and S10) revealed its spectroscopic features were closely similar to those of **1**, suggesting that they possessed the same steroidal nucleus with an *α*,*β*-*α*′,*β*′-unsaturated carbonyl moiety [*δ*_H_ 7.05 (d, *J* = 10.1 Hz, H-1), 6.22 (dd, *J* = 10.1, 1.9 Hz, H-2), 6.06 (br s, H-4), and *δ*_C_ 156.1 (d, C-1), 127.6 (d, C-2), 186.6 (s, C-3), 123.9 (d, C-4), 169.5 (s, C-5)]. In fact, the differences between **2** and **1** were in the structures of their side chains. The carbonyl group shifted from C-22 in **1** to C-23 (*δ*_C_ 215.0) in **2**, and the hydroxyl group attached to C-24 (*δ*_C_ 74.0 vs. *δ*_C_ 53.0) in **1** was lost in **2**, which was consistent with their 16 mass units difference. The characteristic ^1^H–^1^H COSY correlations from H-17 (*δ*_H_ 1.13) through H-20 (*δ*_H_ 2.04) to H_2_-22 (*δ*_H_ 2.20, 2.44) and from H_3_-28 (*δ*_H_ 0.98) through H-24 (*δ*_H_ 2.29) and H-25 (*δ*_H_ 1.92) to H_3_-26 (*δ*_H_ 0.84)/H_3_-27 (*δ*_H_ 0.90), together with the diagnostic HMBC correlations from H_2_-22 to C-20 (*δ*_C_ 32.0) and C-23, from H_3_-28 to C-23, C-24, and C-25 (*δ*_C_ 30.2) (Figure 4, Figures S12 and S13), supported the above-mentioned structure of the side chain. The literature surveys revealed that the NMR data of the side chain of **2** were almost identical to those of the synthetic steroid 3*β*-hydroxyergost-5,7-diene-23-one [36], further confirming the established structure of the side chain. Due to the isomerization of a single isolated chiral center C-24 in a linear chain would not result in significant shifts of ^1^H or ^13^C NMR data, the configuration of C-24 was undetermined. Similar NOE correlations as those of **1** were observed in the NOESY spectrum of **2** (Figure 4 and Figure S14), suggesting they had the same relative configurations for the chiral centers of the parent nucleus. Thus, the structure of **2** was depicted as shown in Figure 1.

**Figure 5:**
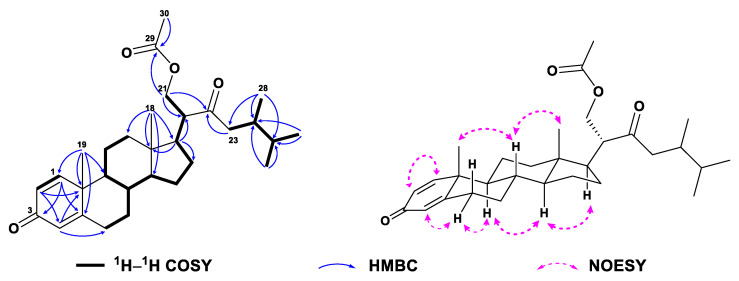
^1^H–^1^H COSY, selected key HMBC and NOE correlations of **3**.

Compound **3**, a white amorphous powder, had the molecular formula of C_30_H_44_O_4_ as established by HRESIMS (Figure S23) from the protonated molecular ion peak observed at *m*/*z* 469.3318 [M + H]^+^ (calcd. 469.3312). Detailed analysis of NMR data of **3** (Table 1 and Table 2, Figures S17 and S18) disclosed that **3** and **1** possessed the same steroidal nucleus but differed in the side chain. The presence of an acetyl group in **3** was recognized by the characteristic NMR signals at *δ*_H_ 2.00 (s, H_3_-30) and *δ*_C_ 170.7 (s, C-29), and 21.0 (q, C-30) (Figure S19). The location of the acetyl group at C-21 was straightforward from the significant down-field shifted NMR signals at *δ*_H_ 4.48 (dd, *J* = 10.7, 4.4 Hz, H_a_-21), 3.96 (t, *J* = 10.7 Hz, H_b_-21), and *δ*_C_ 64.6 (t, C-21), which was further established by the diagnostic HMBC correlations from H_2_-21 to C-17 (*δ*_C_ 49.5), C-20 (*δ*_C_ 53.6), C-22 (*δ*_C_ 211.8), and C-29 (*δ*_C_ 170.7) (Figure 5 and Figure S20). Moreover, the chemical shift of C-24 (*δ*_C_ 38.7) shifted significantly upfield, which indicated that the hydroxyl group attached to C-24 in **1** was lost in **3**. Based on the analysis of the NOE correlations, as depicted in Figure 5 and Figure S22, the structure of **3** was determined, as shown in Figure 1. However, the configuration of C-24 could not be assigned herein.

**Figure 6:**
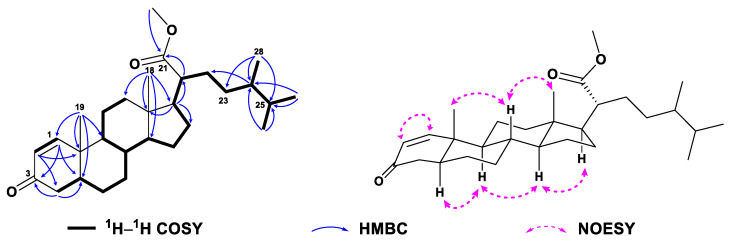
^1^H–^1^H COSY, selected key HMBC and NOE correlations of **4**.

Compound **4** was obtained as a white amorphous powder. Its molecular formula, C_29_H_46_O_3_, was deduced from its protonated molecular ion peak observed at *m*/*z* 443.3520 ([M + H]^+^; calcd. 443.3520) in the HRESIMS spectrum (Figure S31). Careful analysis of its ^1^H and ^13^C NMR data (Table 1 and Table 2, Figures S25 and S26) revealed the presence of an *α*,*β*-unsaturated carbonyl group [*δ*_H_ 7.11 (d, *J* = 10.2 Hz, H-1), 5.84 (dd, *J* = 10.2, 1.0 Hz, H-2), and *δ*_C_ 158.7 (d, C-1), 127.5 (d, C-2), 200.4 (s, C-3)] and a methyl ester functionality [*δ*_H_ 3.65 (s, H_3_-29) and *δ*_C_ 176.9 (s, C-21), 51.2 (s, C-29)] in the molecule. Searching in our compound library, it was found that the ^13^C NMR data of C-1–C-21 were nearly identical to those of methyl spongoate, a steroid previously reported from the soft coral *Spongodes* sp. by our group [37], suggesting they had the same steroidal nucleus and a methoxycarbonyl group at C-21 of the side chain. The only difference between them was at the methyl at C-24 in **4**, which was deduced from the ^1^H–^1^H COSY correlations from H_3_-28 (*δ*_H_ 0.76) through H-24 (*δ*_H_ 1.24) and H-25 (*δ*_H_ 1.55) to H_3_-26 (*δ*_H_ 0.75)/H_3_-27 (*δ*_H_ 0.84) as well as the HMBC correlations from H_3_-28 to C-23 (*δ*_C_ 21.2), C-24 (*δ*_C_ 38.7), and C-25 (*δ*_C_ 31.4) (Figure 6, Figures S28 and S29). The established structure of the side chain was further verified in agreement with the ^13^C NMR data of those of (24*S*)-3*β*-acetoxyergost-5-en-21-oic acid, a secondary metabolite previously reported from the soft coral *Cladiella australis* [38]. Similar NOE correlations as those of methyl spongoate were observed in the ROESY spectrum of **4** (Figure 6 and Figure S30), suggesting they had the same relative configurations for the chiral centers of the parent nucleus. Therefore, compound **4** was established as a 24-methyl derivative of methyl spongoate, as shown in Figure 1, with the configuration of C-24 remaining unknown.

**Figure 7:**
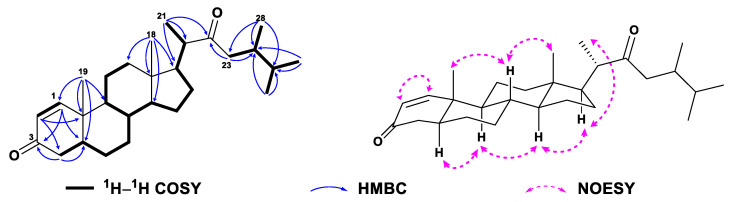
^1^H–^1^H COSY, selected key HMBC and NOE correlations of **5**.

Compound **5** was obtained as a white amorphous powder, and its molecular formula was established as C_28_H_44_O_2_ according to the protonated molecular ion at *m*/*z* 413.3411 ([M + H]^+^; calcd. 413.3414) in the HRESIMS spectrum (Figure S39). A comparison of overall ^1^H and ^13^C NMR data (Table 1 and Table 2, Figures S33 and S34) revealed that **5** shared the identical steroidal nucleus with **4** but differed at the side chain, where the presence of a ketone at C-22 and the disappearance of a methoxycarbonyl group at C-21 were observed. These differences were evident by the NMR signals at *δ*_H_ 1.09 (d, *J* = 6.9 Hz, H_3_-21)/*δ*_C_ 16.7 (q, C-21) and *δ*_C_ 214.8 (s, C-22) (Figure S35). The ^1^H–^1^H COSY correlations from H-17 (*δ*_H_ 1.63) through H-20 (*δ*_H_ 2.50) to H_3_-21 (*δ*_H_ 1.08), together with the HMBC correlations from H_3_-21 to C-17 (*δ*_C_ 52.4), C-20 (*δ*_C_ 49.9), and C-22 (*δ*_C_ 214.8) and from H-23 (*δ*_H_ 2.17) to C-22 and C-24 (*δ*_C_ 38.7) (Figure 6, Figures S36 and S37) supported the speculation. Furthermore, the coincident ^13^C NMR data from C-20 to C-25 and C-28 for **5** and the synthetic steroid 3*β*-hydroxyergost-5,7-diene-22-one [36] confirmed they shared the same side chain. Based on the analysis of the ROESY correlations, as depicted in Figure 7 and Figure S38, the structure of **5** was determined with the unknown configuration of C-24, as shown in Figure 1.

**Figure 8:**
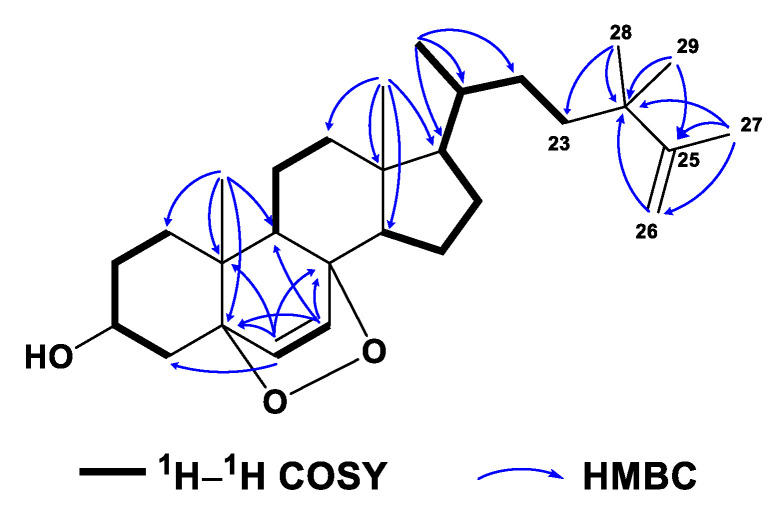
^1^H–^1^H COSY and selected key HMBC correlations of **6**.

Compound **6** was obtained as a white amorphous powder. Its molecular formula C_29_H_46_O_3_ was determined by the HREIMS ion peak at *m*/*z* 424.3325 [M − H_2_O]^+^ (calcd. 424.3336, Figure S47), corresponding to seven degrees of unsaturation. Two vicinal coupled olefinic protons at *δ*_H_ 6.24 (d, *J* = 8.5 Hz, H-6) and 6.50 (d, *J* = 8.6 Hz, H-7) and an oxygenated methine at *δ*_H_ 3.97 (tt, *J* = 11.2, 5.1 Hz, H-3) were characteristic of a 3*β*-hydroxy-6-en-5*α*,8*α*-epidioxysterol nucleus, which was also recognized by the ^13^C NMR signals at *δ*_C_ 66.6 (d, C-3), 82.3 (s, C-5), 135.6 (d, C-6), 130.9 (d, C-7), and 79.6 (s, C-8) (Figure S43). These spectral data of **6** (Table 1 and Table 2, Figures S41 and S42) were reminiscent of yalongsterol A, a sterol previously reported from the soft coral *Sinularia* sp. by our group [11]. Detailed comparison of the full ^1^H and ^13^C NMR data of **6** with yalongsterol A, showing great similarity between them, clearly allowed the assignment of 3*β*-hydroxy-6-en-5*α*,8*α*-epidioxy-cholesta nucleus to **6**, which was further justified by the extensive analyses of 2D NMR spectra involving ^1^H–^1^H COSY, HSQC, and HMBC (Figure 8 and Figures S43–S45). However, they differed at the side chain. The NMR signals at *δ*_H_ 4.72 (br s, H_a_-26), 4.65 (br s, H_b_-26), 1.67 (s, H_3_-27) and *δ*_C_ 152.3 (d, C-25), 109.6 (d, C-26), 19.5 (q, C-27) indicated the presence of a terminal double bound with an allylic methyl in the terminal of the side chain of **6**, which was supported by the HMBC correlations from H_2_-26 (*δ*_H_ 4.65, 4.72) to C-24 (*δ*_C_ 38.8), C-25 (*δ*_C_ 152.3) and C-27 (*δ*_C_ 19.5), H_3_-27 (*δ*_H_ 1.67) to C-24, C-25 and C-26 (*δ*_C_ 109.6) (Figure 8 and Figure S44). Additional HMBC correlations from H_3_-28 (*δ*_H_ 1.00) to C-23 (*δ*_C_ 37.1), C-24, C-25, and C-29 (*δ*_C_ 27.7), from H_3_-29 (*δ*_H_ 1.00) to C-23, C-24, C-25, and C-28 (*δ*_C_ 27.3) (Figure 8 and Figure S44) implied the location of germinal methyls at C-24 of the side chain of **6**. With the established structure of the side chain in hand, the structure of **6** was depicted as shown in Figure 1.

In in vitro bioassays, all the isolates were tested for antibacterial, neuroprotective, and anti-inflammatory effects. In the antibacterial bioassays (Table 3), all the steroids exhibited significant antibacterial activities against the fish pathogenic bacteria *Streptococcus parauberis* FP KSP28, *Phoyobacterium damselae* FP2244, and *Streptococcus parauberis* SPOF3K with IC_90_ values ranging from 0.1 to 11.0 µM. As observed in Table 3, the steroids possessing the unsaturated carbonyl moiety in ring A were favored for the inhibition against *Streptococcus parauberis* FP KSP28. Moreover, it seemed that a vinyl-type side chain in the steroid could lead to a small increase in the antibacterial activities against *Phoyobacterium damselae* FP2244 and *Streptococcus parauberis* SPOF3K, as indicated in Table 3. Only compound **7** displayed potent inhibitory activity against the fish pathogenic bacterium *Aeromonas salmonicida* AS42 with an IC_90_ value of 8.8 µM. This might imply that the combination of an *α*,*β*-*α*′,*β*′-unsaturated carbonyl moiety and a vinyl-type side chain played a key role in the antibacterial activity against *Aeromonas salmonicida* AS42. The above-mentioned results indicated that these isolated steroids could be used as antibacterial agents in fish farming.

Meanwhile, compounds **2** and **6**–**10** also displayed potent inhibitory effects against the vancomycin-resistant *Enterococcus faecium* bacterium G7 with IC_90_ values ranging from 4.4 to 18.3 µM (Table 3). Among them, steroid **10** also displayed antibacterial effects against the vancomycin-resistant *Enterococcus faecium* bacteria G1, G4, and G8 with IC_90_ values of 8.0, 4.0, and 8.0 µM, respectively. The preliminary analysis of the structure-activity relationship for compounds **6**–**10** revealed that the higher degrees of unsaturation of ring A in the steroids could keep efficacy against more individuals of the vancomycin-resistant *Enterococcus faecium* bacteria. The above-mentioned results implied that these isolates could be developed as new chemotypes of antibacterial leads against drug-resistant bacteria.

Moreover, all the isolated steroids except **5**, **6**, and **9** showed strong inhibitory activities against *Streptococcus agalactiae* WR10 with IC_90_ values ranging from 4.0 to 22.0 µM (Table 3). However, in the neuroprotective bioassays, none of these steroids displayed significant neuroprotective effects against the corticosterone-induced cellular injuries in human neuroblastoma SH-SY5Y cells at the concentration of 10 μM. In the evaluations of the anti-inflammatory effect in lipopolysaccharide (LPS)-stimulated BV-2 microglial cells, all the isolates were judged as inactive at 10 μM, neither.

## 3. Materials and Methods

### 3.1. General Experimental Procedures

Optical rotations were measured on a Perkin–Elmer 241 MC polarimeter. The X-ray measurement was made on a Bruker D8 Venture X-ray diffractometer with Cu K*α* radiation (Bruker Biospin AG, Fällanden, Germany). IR spectra were recorded on a Nicolet 6700 spectrometer (Thermo Scientific, Waltham, MA, USA). NMR spectra were measured in CDCl_3_ with a Bruker DRX-400, Bruker DRX-600, or Bruker DRX-800 spectrometer (Bruker Biospin AG, Fällanden, Germany) with the residual CDCl_3_ (*δ*_H_ 7.26 ppm, *δ*_C_ 77.16 ppm). Chemical shifts (*δ*) were reported in ppm with reference to the solvent signals, and coupling constants (*J*) were expressed in Hz. Structural assignments were supported by ^1^H–^1^H COSY, HSQC, HMBC, and NOESY experiments. HREIMS data were recorded on a Finnigan-MAT-95 mass spectrometer (Finnigan-MAT, San Jose, CA, USA). HRESIMS spectra were recorded on an Agilent G6520 Q-TOF mass spectrometer. Commercial silica gel (Qingdao Haiyang Chemical Group Co., Ltd., Qingdao, China, 200–300 and 300–400 mesh) and Sephadex LH-20 gel (Amersham Biosciences, Little Chalfont, UK) were used for column chromatography (CC), and precoated-silica-gel-plates (G60 F-254, Yan Tai Zi Fu Chemical Group Co., Yantai, China) were used for analytical TLC. Reversed-phase (RP) HPLC was performed on an Agilent 1260 series liquid chromatography equipped with a DAD G1315D detector at 210 and 254 nm. A semi-preparative ODS-HG-5 column (5 μm, 250 × 9.4 mm) was employed for the purifications. All solvents used for CC and HPLC were of analytical grade (Shanghai Chemical Reagents Co., Ltd., Shanghai, China) and chromatographic grade (Dikma Technologies Inc., Beijing, China), respectively.

### 3.2. Animal Material

The soft coral *Lobophytum* sp. was collected in October 2021 in Xuwen Country, Guangdong Province, China. This specimen was identified by Prof. X.-B. Li from Hainan University. A voucher specimen (No. S-21-XW-6553) is available for inspection at the Shanghai Institute of Materia Medica, Chinese Academy of Sciences.

### 3.3. Extraction and Isolation

The frozen animals (1275 g, dry weight) were cut into pieces and extracted exhaustively with acetone at room temperature (4 × 3.0 L, 15 min in ultrasonic bath). The organic extract was evaporated to give a brown residue, which was then partitioned between Et_2_O and H_2_O. The Et_2_O solution was concentrated under reduced pressure to give a dark brown residue (13.4 g), which was fractionated by gradient Si gel (200–300 mesh) column chromatography (CC) (Et_2_O/petroleum ether (PE), 0→100%), yielding eight fractions (A–H). Fraction C was chromatographed over Sephadex LH-20 CC (PE/CH_2_Cl_2_/MeOH, 2:1:1) to give compound **10** (1.5 mg) and a mixture. This mixture was further purified through a silica gel CC (300–400 mesh, PE:Et_2_O, 12:1) followed by RP-HPLC (80% MeOH, 0.8 mL/min) to afford compound **6** (3.8 mg, *t*_R_ = 31.3 min). Fraction D was subjected to a column of Sephadex LH-20 eluted with PE/CH_2_Cl_2_/MeOH (2:1:1) to yield three subfractions (D1–D3). Compound **8** (1.5 mg, *t*_R_ = 20.0 min) was obtained from the D1 through a silica gel CC (300–400 mesh, PE:Et_2_O, 10:1) followed by RP-HPLC (85% CH_3_CN, 1.0 mL/min). Compound **9** (2.0 mg, *t*_R_ = 29.1 min) was isolated from the D2 through silica gel CC (300–400 mesh, PE:Et_2_O, 10:1) followed by RP-HPLC (85% CH_3_CN, 1.0 mL/min). Compounds **4** (1.7 mg, *t*_R_ = 25.9 min) and **5** (0.8 mg, *t*_R_ = 22.0 min) were obtained from the D3 through silica gel CC (300–400 mesh, PE:Et_2_O, 10:1) followed by RP-HPLC (77% CH_3_CN, 1.0 mL/min). Fraction E was subjected to Sephadex LH-20 CC (PE/CH_2_Cl_2_/MeOH, 2:1:1), followed by RP-HPLC (75% CH_3_CN, 0.6 mL/min) to give compound **7** (3.1 mg, *t*_R_ = 25.8 min). Fraction F was subjected to a column of Sephadex LH-20 eluted with PE/CH_2_Cl_2_/MeOH (2:1:1) and further divided into two subfractions, F1 and F2, by the following silica gel CC (300–400 mesh, PE:Et_2_O, 3:1). Compounds **1** (2.6 mg, *t*_R_ = 8.7 min) and **3** (1.1 mg, *t*_R_ = 12.6 min) were obtained from F2 by RP-HPLC (65% CH_3_CN, 0.8 mL/min) while **2** (1.6 mg, *t*_R_ = 28.0 min) was obtained from F1 by RP-HPLC (75% CH_3_CN, 0.8 mL/min).

### 3.4. Spectroscopic Data of Compounds

Lobosteroid A (**1**): Colorless crystal; [α${]}_{20}^{D}$ −3.8 (c 0.26, CHCl_3_); IR (KBr): *ν*_max_ 3358, 2922, 2851, 1661, 1632, 1468, 1180 cm^−1^; ^1^H and ^13^C NMR (CDCl_3_, 800 and 125 MHz; see Table 1 and Table 2); HRESIMS *m*/*z* 427.3210 [M + H]^+^ (calcd. for C_28_H_43_O_3_, 427.3207).

Lobosteroid B (**2**): White amorphous powder; [α${]}_{20}^{D}$ +14.0 (c 0.16, CHCl_3_); IR (KBr): *ν*_max_ 3358, 2925, 2852, 1664, 1631, 1467, 887 cm^−1^; ^1^H and ^13^C NMR (CDCl_3_, 600 and 150 MHz; see Table 1 and Table 2); HRESIMS *m*/*z* 411.3255 [M + H]^+^ (calcd. for C_28_H_43_O_2_, 411.3258).

Lobosteroid C (**3**): White amorphous powder; [α${]}_{20}^{D}$ +18.2 (c 0.05, CH_3_OH); IR (KBr): *ν*_max_ 3359, 2923, 2852, 1742, 1662, 1468, 1236 cm^−1^; ^1^H and ^13^C NMR (CDCl_3_, 600 and 150 MHz; see Table 1 and Table 2); HRESIMS *m*/*z* 469.3318 [M + H]^+^ (calcd. for C_30_H_45_O_4_, 469.3312).

Lobosteroid D (**4**): White amorphous powder; [α${]}_{20}^{D}$ +9.2 (c 0.17, CHCl_3_); IR (KBr): *ν*_max_ 3359, 2923, 2852, 1660, 1633, 1468 cm^−1^; ^1^H and ^13^C NMR (CDCl3, 600 and 200 MHz; see Table 1 and Table 2); HRESIMS *m*/*z* 443.3520 [M + H]^+^ (calcd. for C_29_H_47_O_3_, 443.3520).

Lobosteroid E (**5**): White amorphous powder; [α${]}_{20}^{D}$ +11.9 (c 0.08, CHCl_3_); IR (KBr): *ν*_max_ 3358, 2922, 2851, 1660, 1633, 1468 cm^−1^; ^1^H and ^13^C NMR (CDCl3, 600 and 200 MHz; see Table 1 and Table 2); HRESIMS *m*/*z* 413.3411 [M + H]^+^ (calcd. for C_28_H_45_O_2_, 413.3414).

Lobosteroid F (**6**): White amorphous powder; [α${]}_{20}^{D}$ −7.9 (c 0.38, CHCl_3_); IR (KBr): *ν*_max_ 3300, 2949, 2869, 1455, 1377, 1044 cm^−1^; ^1^H and ^13^C NMR (CDCl_3_, 400 and 150 MHz; see Table 1 and Table 2); HREIMS *m*/*z* 424.3325 [M − H_2_O]^+^ (calcd. for C_29_H_44_O_2_, 424.3336).

### 3.5. X-ray Crystallographic Analysis for Compound ***1***

Lobosteroid A (**1**) was crystallized from MeOH at room temperature. C_28_H_43_O_3_, Mr = 426.61, monoclinic, crystal size 0.12 × 0.08 × 0.05 mm^3^, space group *P*2_1_2_1_2_1_, a = 11.7623(13) Å, b = 11.875(2) Å, c = 17.1256(15) Å, V = 2392.0(6) Å^3^, Z = 4, ρ_calcd_ = 1.185 g/cm^3^, F(000) = 936.0, 31,225 collected reflections, 4920 independent reflections (R_int_ = 0.0522, R_sigma_ = 0.0310), final R1 = 0.0353 (wR_2_ = 0.0904) reflections with I ≥ 2σ (I), R_1_ = 0.0383, wR_2_ = 0.0951 for all unique data. The X-ray measurements were made on a Bruker D8 Venture X-ray diffractometer with Cu K*α* radiation (*λ* = 1.54178 Å) at 170.0 K. The collected data integration and reduction were processed with SAINT V8.37A software, and multiscan absorption corrections were performed using the SADABS program. The structure was solved with the SHELXT [39] structure solution program using intrinsic phasing and refined with the SHELXL [40] refinement package using least squares minimization. Crystallographic data for **1** were deposited at the Cambridge Crystallographic Data Centre (Deposition nos. CCDC 2282723). Copies of these data can be obtained free of charge via www.ccdc.cam.ac.uk (accessed on 19 July 2023), or from the Cambridge Crystallographic Data Centre, 12 Union Road, Cambridge CB21EZ, UK. [Fax: (+44) 1223-336-033. E-mail: deposit@ccdc.cam.ac.uk.]

### 3.6. Antibacterial Bioassays

The marine strains *Streptococcus parauberis* FP KSP28, *Streptococcus parauberis* SPOF3K, *Phoyoba cteriumdamselae* FP2244, and *Aeromonas salmonicida* AS42 were provided by National Fisheries Research & Development Institute, Korea. The strain *Streptococcus agalactiae* WR10 was provided by the Chinese Academy of Tropical Agricultural Sciences. The vancomycin-resistant *Enterococcus faecium* bacteria G1, G4, G7, and G8 were provided by Ruijin Hospital, Shanghai Jiao Tong University School of Medicine. The minimum inhibitory concentration for 90% (MIC_90_) values for all antimicrobial agents was measured by the 96-well micro-dilution method. Mueller–Hinton II broth (cation-adjusted, BD 212322) was used for MIC_90_ value determination. Generally, compounds were dissolved with DMSO to 20 mM as stock solutions. All samples were diluted with culture broth to 500 µM as the initial concentration. Further, 1:2 serial dilutions were performed by the addition of culture broth to reach concentrations ranging from 500 µM to 0.24 µM. 100 µL of each dilution was distributed in 96-well plates, as well as sterile controls, growth controls (containing culture broth plus DMSO, without compounds), and positive controls (containing culture broth plus control antibiotics such as tetracycline). Each test and growth control well was inoculated with 5 µL of an exponential-phase bacterial suspension (about 10^5^ CFU/well). The 96-well plates were incubated at 37 °C for 24 h. MIC_90_ values of these compounds were defined as the lowest concentration to inhibit bacterial growth completely. All MIC_90_ values were interpreted according to the recommendations of the Clinical and Laboratory Standards Institute (CLSI). Tetracycline hydrochloride (TC), oxytetracycline hydrochloride (OT), and levofloxacin hydrochloride (LF) were used as positive controls (Table 3).

## 4. Conclusions

In summary, six new steroids, lobosteroids A–F (**1**–**6**), together with four known compounds **7**–**10**, were isolated from the Chinese soft coral *Lobophytum* sp. The chemical diversity of new steroids was mainly attributed to the high oxidation, which was characterized by the conjugated enone or dienone system of the nucleus and diverse oxidation of side chains. Although many steroids were reported from soft corals, those with an *α*,*β*-*α*′,*β*′-unsaturated carbonyl or an *α*,*β*-unsaturated carbonyl moiety in ring A, or the existence of a 5*α*,8*α*-epidioxy system in ring B were rarely found from the genus *Lobophytum*. The discovery of steroids **1**–**6** expanded the diverse and complex array of steroids, which is still a research hotspot of marine natural products. In the bioassays, all of the isolates displayed significant antibacterial activities against the fish pathogenic bacteria *Streptococcus parauberis* FP KSP28, *Phoyobacterium damselae* FP2244, and *Streptococcus parauberis* SPOF3K with IC_90_ values ranging from 0.1 to 11.0 µM. Meanwhile, compounds **2** and **6**–**10** exhibited excellent antibacterial activities against the vancomycin-resistant *Enterococcus faecium* bacterium G7 with IC_90_ values ranging from 4.4 to 18.3 µM. These new findings implied that these isolated steroids could be developed as new chemotypes of antibacterial leads.

## Figures and Tables

**Table 1:** ^13^C NMR data of compounds **1**–**6** in CDCl_3_.

No.	1 ^a^	2 ^b^	3 ^a^	4 ^a^	5 ^a^	6 ^c^
	*δ*_C_ (Mult.)	*δ*_C_ (Mult.)	*δ*_C_ (Mult.)	*δ*_C_ (Mult.)	*δ*_C_ (Mult.)	δC (Mult.)
1	155.8 (d)	156.2 (d)	155.7 (d)	158.7 (d)	158.6 (d)	34.8 (t)
2	127.7 (d)	127.6 (d)	127.8 (d)	127.5 (d)	127.6 (d)	30.3 (t)
3	186.5 (s)	186.6 (s)	186.5 (s)	200.4 (s)	200.4 (s)	66.6 (d)
4	124.1 (d)	123.9 (d)	124.1 (d)	41.1 (t)	41.1 (t)	37.1 (d)
5	169.1 (s)	169.5 (s)	169.0 (s)	44.4 (d)	44.4 (d)	82.3 (s)
6	33.0 (t)	33.0 (t)	32.9 (t)	27.7 (t)	27.7 (t)	135.6 (d)
7	33.7 (t)	33.8 (t)	33.6 (t)	32.1 (t)	31.4 (t)	130.9 (d)
8	35.6 (d)	35.6 (d)	35.6 (d)	35.8 (d)	35.8 (d)	79.6 (s)
9	52.3 (d)	52.4 (d)	52.2 (d)	50.1 (d)	49.9 (d)	51.2 (d)
10	43.6 (s)	43.8 (s)	43.6 (s)	39.1 (s)	39.1 (s)	37.1 (s)
11	22.9 (t)	24.5 (t)	22.8 (t)	30.1 (t)	21.3 (t)	23.5 (t)
12	39.5 (t)	39.5 (t)	38.8 (t)	37.5 (t)	39.8 (t)	39.5 (t)
13	43.1 (s)	42.9 (s)	43.0 (s)	42.4 (s)	43.1 (s)	44.8 (s)
14	54.9 (d)	55.6 (d)	54.6 (d)	55.8 (d)	55.8 (d)	51.7 (d)
15	24.7 (t)	23.0 (t)	24.5 (t)	23.8 (t)	24.5 (t)	20.8 (t)
16	27.5 (t)	28.4 (t)	26.9 (t)	27.3 (t)	27.7 (t)	28.3 (t)
17	52.0 (d)	56.0 (d)	49.6 (d)	52.9 (d)	52.4 (d)	56.2 (d)
18	12.4 (q)	12.2 (q)	12.5 (q)	12.4 (q)	12.6 (q)	12.7 (q)
19	18.8 (q)	18.8 (q)	18.8 (q)	13.1 (q)	13.1 (q)	18.3 (q)
20	50.9 (d)	32.0 (d)	53.6 (d)	48.0 (d)	50.0 (d)	35.8 (d)
21	16.4 (q)	20.0 (q)	64.6 (t)	176.9 (s)	16.7 (q)	18.9 (q)
22	217.6 (s)	49.2 (t)	211.8 (s)	29.8 (t)	214.8 (s)	30.4 (t)
23	48.2 (t)	215.0 (s)	49.6 (t)	21.2 (t)	46.8 (t)	37.1 (t)
24	74.0 (s)	53.0 (d)	33.3 (d)	38.7 (d)	33.8 (d)	38.8 (s)
25	37.4 (d)	30.2 (d)	32.1 (d)	31.4 (d)	32.1 (d)	152.3 (s)
26	17.0 (q)	18.8 (q)	18.6 (q)	17.5 (q)	18.3 (q)	109.6 (t)
27	17.9 (q)	21.6 (q)	19.9 (q)	20.6 (q)	20.0 (q)	19.5 (q)
28	23.0 (q)	12.7 (q)	16.2 (q)	15.3 (q)	16.1 (q)	27.3 (q)
29			170.7 (s)	51.2 (q)		27.7 (q)
30			21.0 (q)			

^a^ Recorded at 125 MHz. ^b^ Recorded at 150 MHz. ^c^ Recorded at 200 MHz.

**Table 2:** ^1^H NMR data of compounds **1**–**6** in CDCl_3_.

No.	1 ^a^	2 ^b^	3 ^a^	4 ^a^	5 ^a^	6 ^c^
	*δ*H Mult. (*J* in Hz)	*δ*H Mult. (*J* in Hz)	*δ*H Mult. (*J* in Hz)	*δ*H Mult. (*J* in Hz)	*δ*H Mult. (*J* in Hz)	*δ*H Mult. (*J* in Hz)
1	7.04 d (10.2)	7.05 d (10.1)	7.03 d (10.1)	7.11 d (10.2)	7.13 d (10.2)	1.70 br d (13.8)
						1.94 dd (13.8, 4.0)
2	6.23 dd (10.2, 2.0)	6.22 dd (10.1, 1.9)	6.23 dd (10.1, 1.9)	5.84 dd (10.2, 1.0)	5.85 d (10.2)	1.54 ovl
						1.84 br d (12.8)
3						3.97 tt (11.2, 5.1)
4	6.07 br ^d^ s	6.06 br s	6.07 br s	2.21 dd (14.5, 3.6)	2.23 dd (14.9, 3.3)	1.91 ovl
				2.35 dd (17.7, 14.2)	2.36 dd (17.8, 14.2)	2.11 dd (13.7, 5.2)
5				1.90 ovl	1.91 ovl	
6	2.35 br d (13.5)	2.36 br d (13.4)	2.35 br d (13.0)	1.37 m	1.31 ovl	6.24 d (8.5)
	2.45 dd (14.0, 5.2)	2.45 ovl	2.46 dd (13.6, 4.0)	1.42 ovl	1.42 ovl	
7	1.93 m	1.03 ovl	1.06 ovl	0.96 ovl	0.96 m	6.50 d (8.6)
	2.48 dd (12.0, 5.0)	1.94 ovl	1.94 ovl	1.70 ovl	1.71 ovl	
8	1.61 ovl ^d^	1.62 ovl	1.61 m	1.45 ovl	1.45 ovl	
9	1.59 ovl	1.04 ovl	1.06 ovl	0.96 ovl	0.99 m	1.49 ovl
11	1.07 m	1.15 ovl	1.66 ovl	0.85 dd (13.1, 6.0)	0.88 m	1.20 ovl
	1.69 ovl	1.61 ovl	1.71 m	1.72 dd (13.3, 3.2)	1.76 dd (13.7, 3.5)	1.50 ovl
12	1.28 td (13.0, 5.0)	1.17 dd (12.5, 6.6)	1.23 ovl	1.07 ovl	1.31 ovl	1.21 ovl
	1.97 dt (13.1, 3.3)	2.04 dt (13.0, 3.3)	1.90 dt (12.6, 3.3)	1.50 ovl	1.97 dt (12.7, 3.4)	1.96 dd (13.3, 3.3)
14	1.04 m	1.00 m	1.01 m	1.08 ovl	1.09 ovl	1.53 ovl
15	1.18 ovl	1.62 ovl	1.20 ovl	1.09 ovl	1.11 ovl	0.89 m
	1.63 td (13.0, 3.5)	1.68 td (11.1, 3.7)	1.64 ovl	1.64 ovl	1.60 ovl	1.64 m
16	1.18 ovl	1.25 m	1.30 m	1.30 ovl	1.32 ovl	1.34 m
	1.73 ovl	1.79 ddd (16.2, 7.0, 3.1)	1.65 ovl	1.90 ovl	1.69 ovl	1.90 ovl
17	1.60 ovl	1.13 m	1.60 ovl	1.65 ovl	1.63 ovl	1.18 ovl
18	0.76 s	0.78 s	0.81 s	0.72 s	0.72 s	0.78 s
19	1.23 s	1.23 s	1.23 s	0.99 s	1.01 s	0.89 s
20	2.48 ovl	2.03 ovl	2.84 td (10.4, 4.4)	2.20 dt (7.5, 3.6)	2.50 dq (10.4. 6.8)	1.32 m
21	1.11 d (7.6)	0.90 d (7.0)	3.96 t (10.7)		1.09 d (6.9)	0.89 d (6.9)
			4.48 dd (10.7, 4.4)			
22		2.20 dd (17.0, 10.0)		1.27 ovl		1.17 ovl
		2.45 dd (17.2, 2.8)		1.40 ovl		1.26 m
23	2.52 d (17.7)		2.22 dd (17.7, 9.1)	1.39 ovl	2.17 dd (16.9, 8.9)	1.17 ovl
	2.66 d (17.7)		2.47 dd (17.5, 3.8)	1.51 ovl	2.45 dd (17.0, 4.3)	1.37 m
24		2.29 quin (6.9)	1.94 ovl	1.24 ovl	1.93 ovl	
25	1.75 quin (6.9)	1.92 ovl	1.55 m	1.55 m	1.55 m	
26	0.88 d (6.9)	0.84 d (6.8)	0.83 d (7.1)	0.75 d (6.9)	0.82 d (6.8)	4.65 br s
						4.72 br s
27	0.93 d (6.8)	0.90 d (7.0)	0.87 d (6.8)	0.84 d (6.8)	0.87 d (6.8)	1.67 s
28	1.12 s	0.98 d (6.9)	0.81 d (7.6)	0.76 d (6.9)	0.81 d (6.8)	1.00 s
29				3.65 s		1.00 s
30			2.00 s			
OH	4.09 s					

^a^ Recorded at 600 MHz. ^b^ Recorded at 800 MHz. ^c^ Recorded at 400 MHz. ^d^ ovl: overlapped, br: broad.

**Table 3:** The IC_90_ values (μM) of antibacterial bioassays of compounds **1**–**10** ^a^.

	1	2	3	4	5	6	7	8	9	10	TC	OT	LF
*Streptococcus parauberis* FP KSP28	0.3	0.5	2.7	0.1	4.9	1.1	0.5	8.9	4.4	1.0	6.3	3.1	3.1
*Phoyobacterium damselae* FP2244	9.1	4.2	11.0	8.5	9.8	9.2	2.2	2.2	4.4	2.0	0.1	0.1	0.1
*Streptococcus parauberis* SPOF3K	9.1	4.2	11.0	8.5	9.8	4.6	2.2	2.2	4.4	2.0	>50.0	25.0	3.1
*Aeromonas salmonicida* AS42	- ^b^	-	-	-	-	-	8.8	-	-	-	12.7	0.8	0.8
*Enterococcus faecium* G1	-	-	-	-	-	-	8.8	-	-	8.0	0.2	0.4	>100.0
*Enterococcus faecium* G4	-	-	-	-	-	-	-	-	-	4.0	0.4	0.4	>100.0
*Enterococcus faecium* G7	-	16.8	-	-	-	18.3	8.8	8.9	4.4	8.0	0.2	0.2	>100.0
*Enterococcus faecium* G8	-	-	-	-	-	-	-	-	-	8.0	0.2	0.1	>100.0
*Streptococcus agalactiae* WR10	18.1	8.4	22.0	17.0	-	-	4.4	4.4	-	4.0	1.6	1.6	6.3

^a^ Tetracycline hydrochloride (TC), oxytetracycline hydrochloride (OT), levofloxacin hydrochloride (LF) were used as positive controls. ^b^ ‘-’ indicated they were not subjected to the antibacterial rescreening experiments since their inhibition rates against these bacteria were <90% in the preliminary antibacterial bioassays.

## Data Availability

Data are contained within the article or Supplementary Materials.

## Supplementary Materials

The following supporting information can be downloaded at: https://www.mdpi.com/article/10.3390/md21080457/s1, Figures S1–S48: NMR, HRESIMS, HREIMS, and IR data of compounds **1**–**6**.
