# Supplementary material for: Lobosteroids A–F: Six New Highly Oxidized Steroids from the Chinese Soft Coral Lobophytum sp
Source: Mar Drugs. 2023 Aug 19;21(8):457. doi: 10.3390/md21080457 (PMC10456462; doi:10.3390/md21080457)
Supplement: Supplementary file 1 [file marinedrugs-21-00457-s001.zip › marinedrugs-2551577-supplementary.pdf]

Supplementary Materials

Figure S1. ^{1}H NMR spectrum (600 MHz) of compound **1** in $CDCl_3$.
Figure S2. ^{13}C NMR spectrum (125 MHz) of compound **1** in $CDCl_3$.
Figure S3. HSQC spectrum (600 MHz) of compound **1** in $CDCl_3$.
Figure S4. HMBC spectrum (600 MHz) of compound **1** in $CDCl_3$.
Figure S5. ^{1}H-^{1}H COSY spectrum (600 MHz) of compound **1** in $CDCl_3$.
Figure S6. NOESY spectrum (600 MHz) of compound **1** in $CDCl_3$.
Figure S7. HR-ESIMS spectrum of compound **1**.
Figure S8. IR spectrum of compound **1**.
Figure S9. ^{1}H NMR spectrum (800 MHz) of compound **2** in $CDCl_3$.
Figure S10. ^{13}C NMR spectrum (150 MHz) of compound **2** in $CDCl_3$.
Figure S11. HSQC spectrum (800 MHz) of compound **2** in $CDCl_3$.
Figure S12. HMBC spectrum (800 MHz) of compound **2** in $CDCl_3$.
Figure S13. ^{1}H-^{1}H COSY spectrum (800 MHz) of compound **2** in $CDCl_3$.
Figure S14. NOESY spectrum (800 MHz) of compound **2** in $CDCl_3$.
Figure S15. HR-ESIMS spectrum of compound **2**.
Figure S16. IR spectrum of compound **2**.
Figure S17. ^{1}H NMR spectrum (600 MHz) of compound **3** in $CDCl_3$.
Figure S18. ^{13}C NMR spectrum (150 MHz) of compound **3** in $CDCl_3$.
Figure S19. HSQC spectrum (600 MHz) of compound **3** in $CDCl_3$.
Figure S20. HMBC spectrum (600 MHz) of compound **3** in $CDCl_3$.
Figure S21. ^{1}H-^{1}H COSY spectrum (600 MHz) of compound **3** in $CDCl_3$.
Figure S22. NOESY spectrum (600 MHz) of compound **3** in $CDCl_3$.
Figure S23. HR-ESIMS spectrum of compound **3**.
Figure S24. IR spectrum of compound **3**.
Figure S25. ^{1}H NMR spectrum (600 MHz) of compound **4** in $CDCl_3$.
Figure S26. ^{13}C NMR spectrum (200 MHz) of compound **4** in $CDCl_3$.
Figure S27. HSQC spectrum (600 MHz) of compound **4** in $CDCl_3$.
Figure S28. HMBC spectrum (600 MHz) of compound **4** in $CDCl_3$.
Figure S29. ^{1}H-^{1}H COSY spectrum (600 MHz) of compound **4** in $CDCl_3$.
Figure S30. ROESY spectrum (600 MHz) of compound **4** in $CDCl_3$.
Figure S31. HR-ESIMS spectrum of compound **4**.
Figure S32. IR spectrum of compound **4**.
Figure S33. ^{1}H NMR spectrum (600 MHz) of compound **5** in $CDCl_3$.
Figure S34. ^{13}C NMR spectrum (200 MHz) of compound **5** in $CDCl_3$.
Figure S35. HSQC spectrum (600 MHz) of compound **5** in $CDCl_3$.
Figure S36. HMBC spectrum (600 MHz) of compound **5** in $CDCl_3$.
Figure S37. ^{1}H-^{1}H COSY spectrum (600 MHz) of compound **5** in $CDCl_3$.
Figure S38. ROESY spectrum (600 MHz) of compound **5** in $CDCl_3$.
Figure S39. HR-ESIMS spectrum of compound **5**.
Figure S40. IR spectrum of compound **5**.
Figure S41. ^{1}H NMR spectrum (400 MHz) of compound **6** in $CDCl_3$.

Figure S42. ^{13}C NMR spectrum (150 MHz) of compound **6** in $CDCl_3$.
Figure S43. HSQC spectrum (600 MHz) of compound **6** in $CDCl_3$.
Figure S44. HMBC spectrum (600 MHz) of compound **6** in $CDCl_3$.
Figure S45. ^{1}H-^{1}H COSY spectrum (600 MHz) of compound **6** in $CDCl_3$.
Figure S46. NOESY spectrum (600 MHz) of compound **6** in $CDCl_3$.
Figure S47. HR-EIMS spectrum of compound **6**.
Figure S48. IR spectrum of compound **6**.

1. Spectra of compound 1

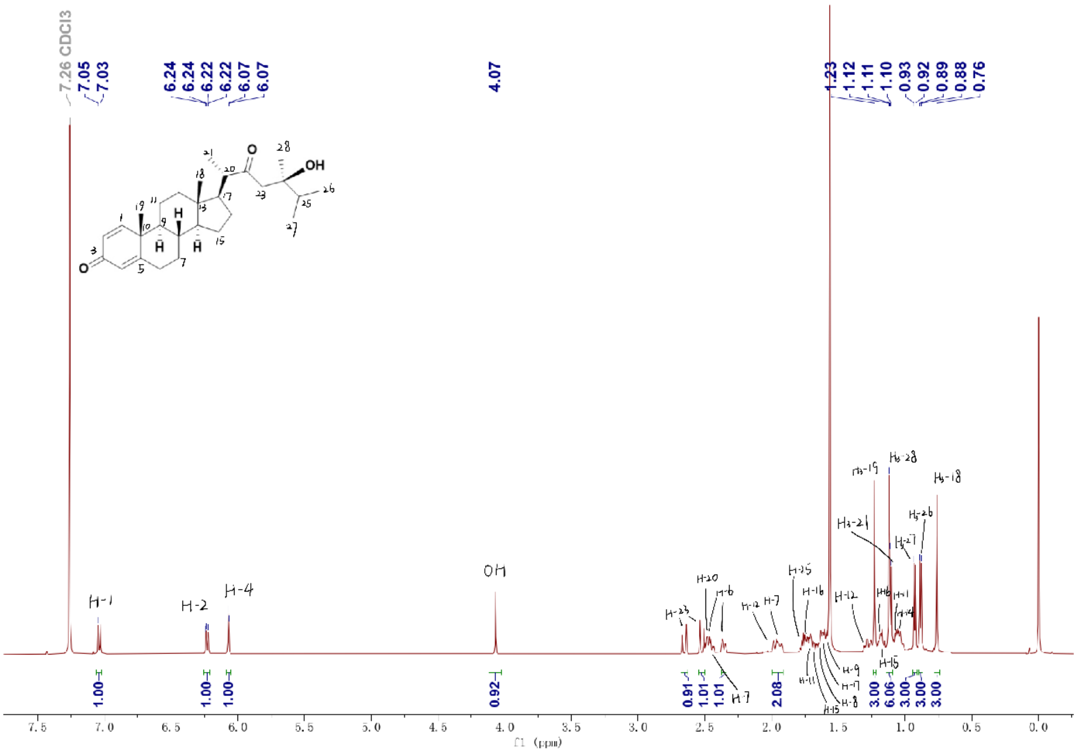

Figure S1. ^{1}H NMR spectrum (600 MHz) of compound **1** in $CDCl_3$.

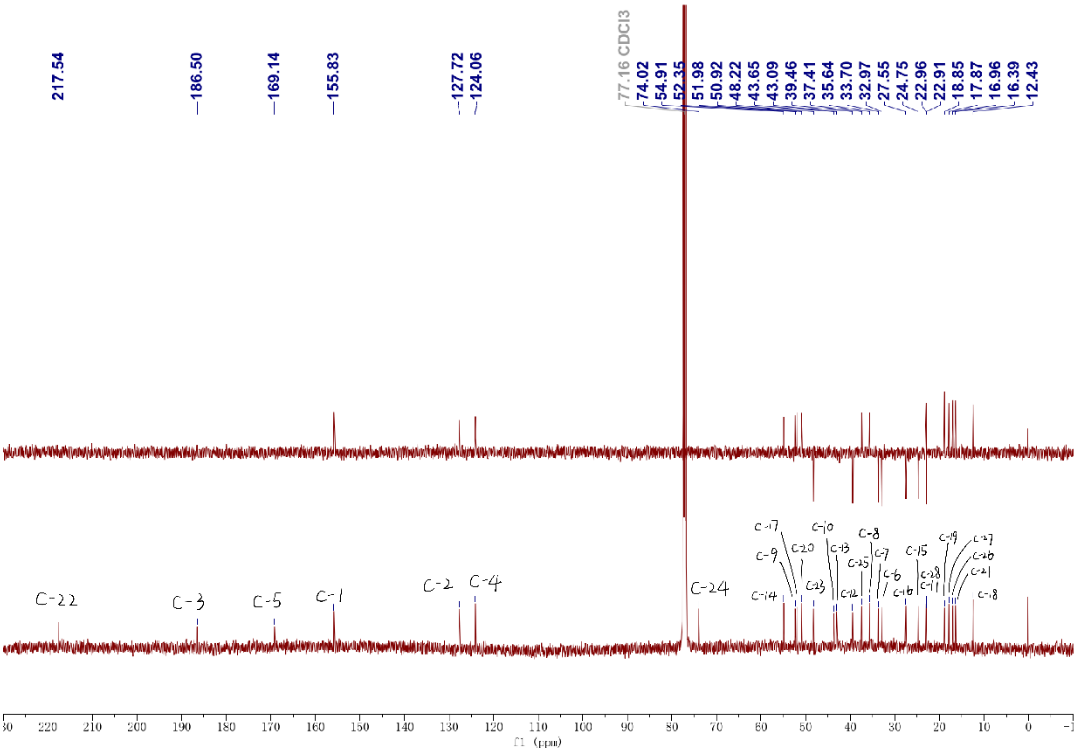

Figure S2. ^{13}C NMR spectrum (125 MHz) of compound **1** in $CDCl_3$.

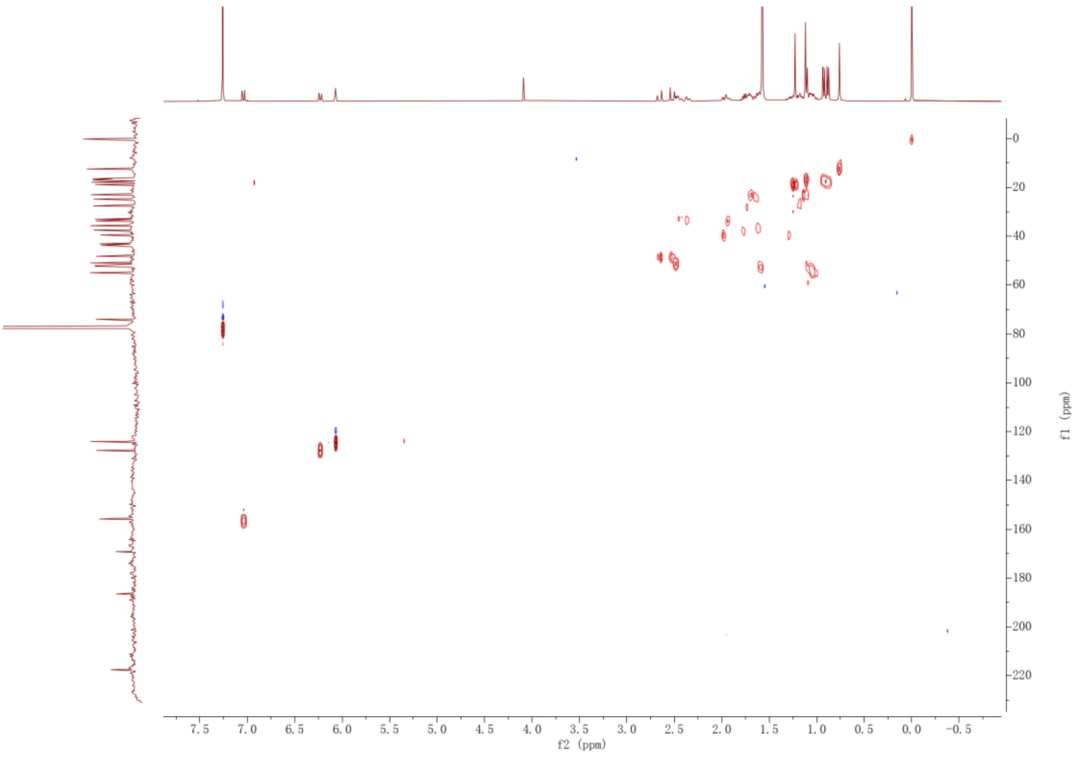

Figure S3. HSQC spectrum (600 MHz) of compound **1** in $CDCl_3$.

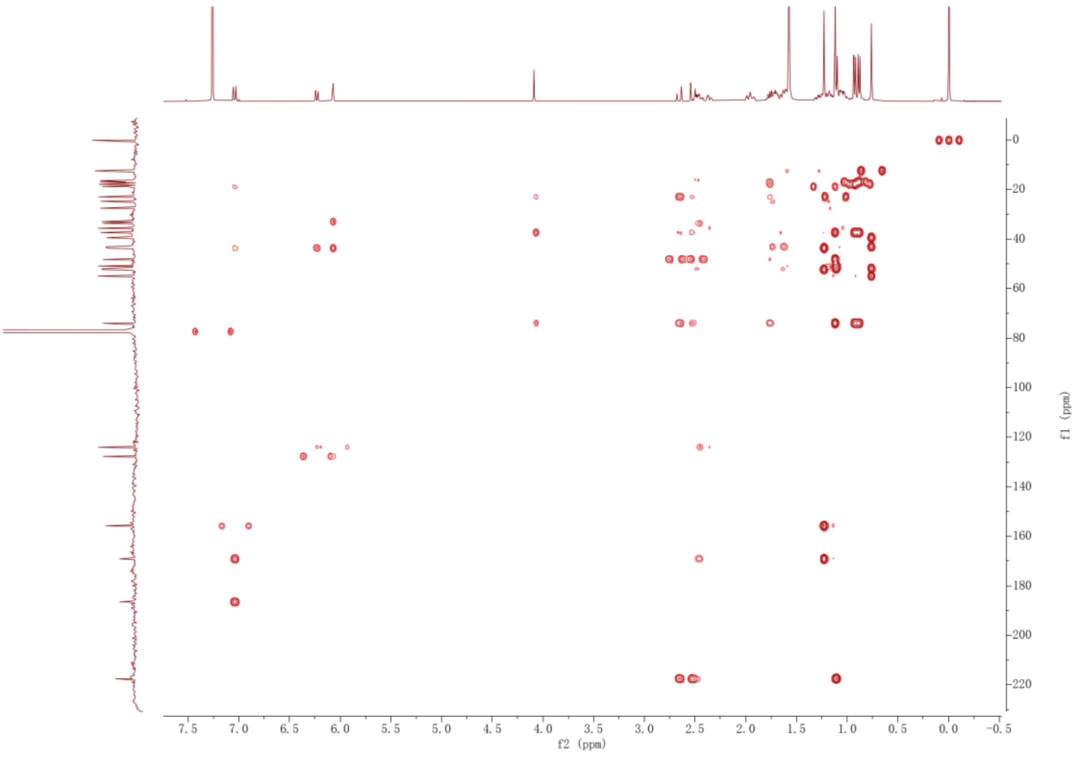

Figure S4. HMBC spectrum (600 MHz) of compound **1** in $CDCl_3$.

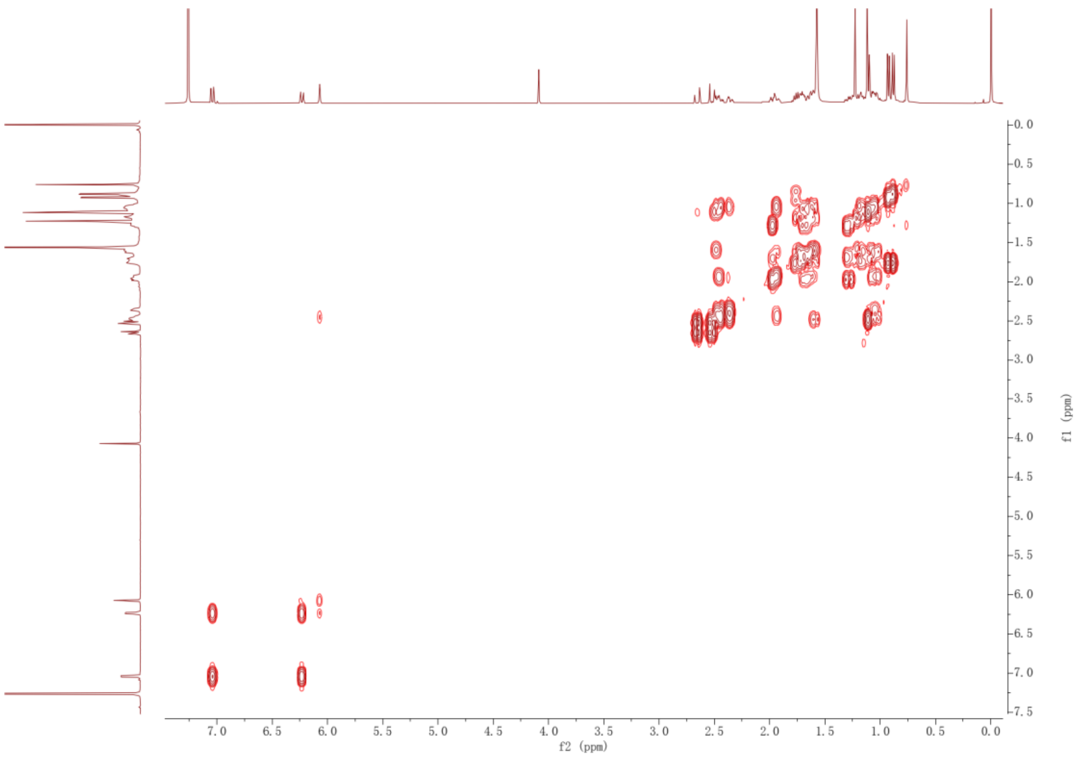

Figure S5. ^{1}H-^{1}H COSY spectrum (600 MHz) of compound **1** in $CDCl_3$.

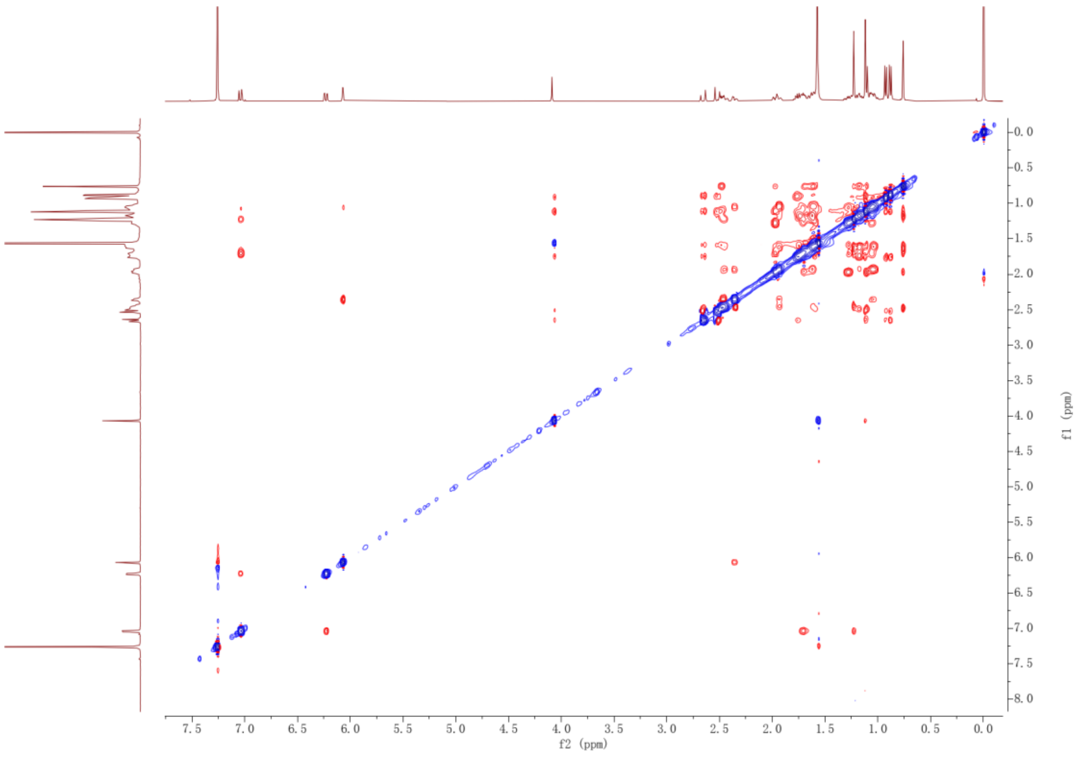

Figure S6. NOESY spectrum (600 MHz) of compound **1** in $CDCl_3$.

**Data Filename**	ESIH202300411.d	**Sample Name**	A8-A8-AFBBBE-4
**Sample ID**		**Position**	P1-D8
**Instrument Name**	Agilent G6520 Q-TOF	**Acq Method**	20160322_MS_ESIH_POS_1min.m
**Acquired Time**	1/11/2023 14:00:02	**IRM Calibration Status**	Success
**DA Method**	small molecular data analysis method.m	**Comment**	ESIH by fangsu

User Spectra

Fragmentor Voltage	Collision Energy	Ionization Mode
170	0	ESI

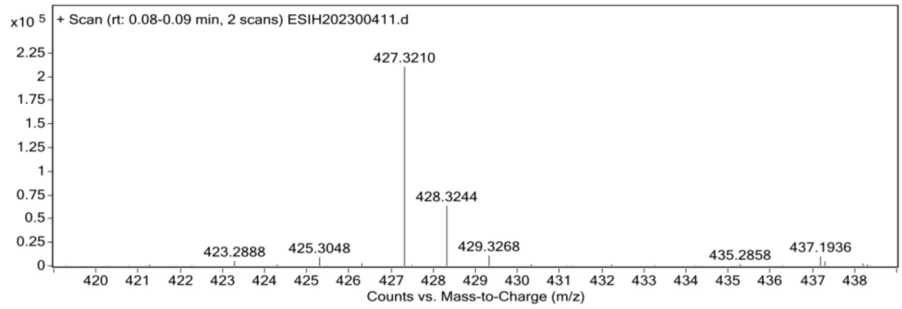

Formula Calculator Results

m/z	Calc m/z	Diff (mDa)	Diff (ppm)	Ion Formula	Ion
427.321	427.3207	-0.3	-0.7	C28 H43 O3	(M+H)+

Figure S7. HR-ESIMS spectrum of compound **1**.

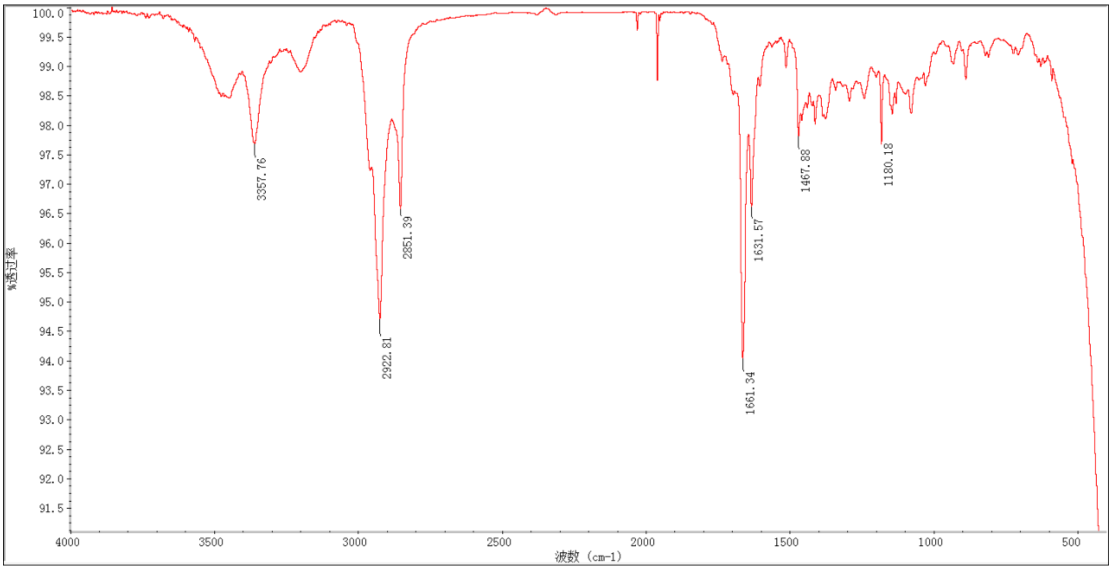

Figure S8. IR spectrum of compound **1**.

2. Spectra of compound 2

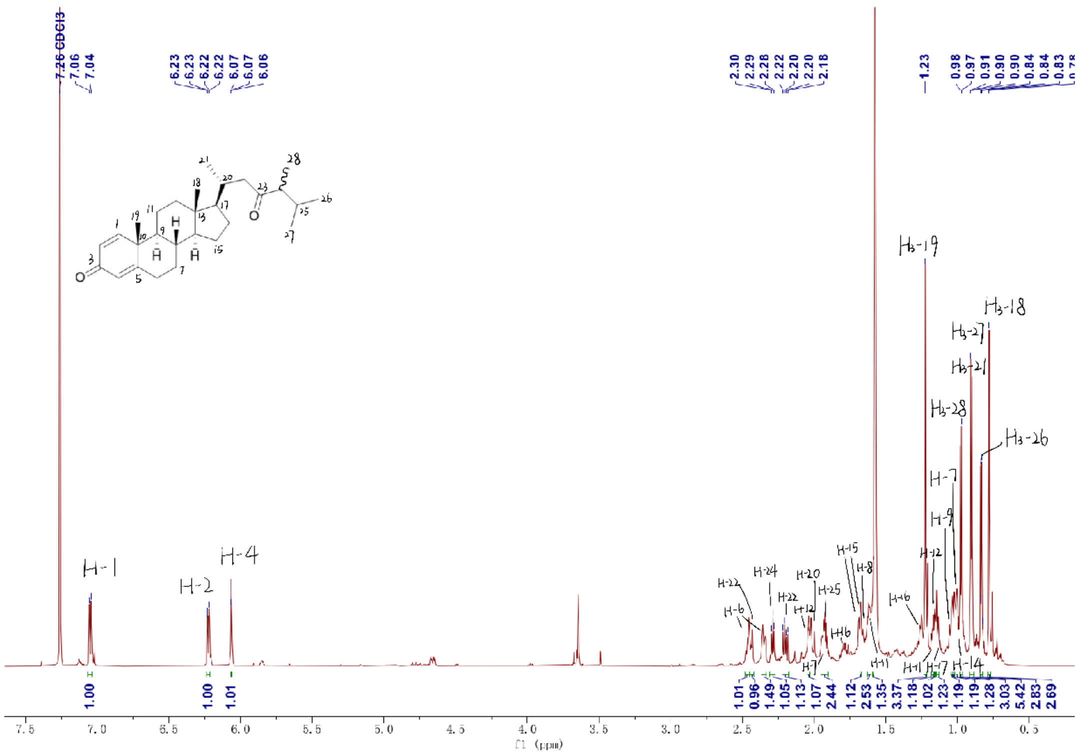

Figure S9. ^{1}H NMR spectrum (800 MHz) of compound **2** in $CDCl_3$.

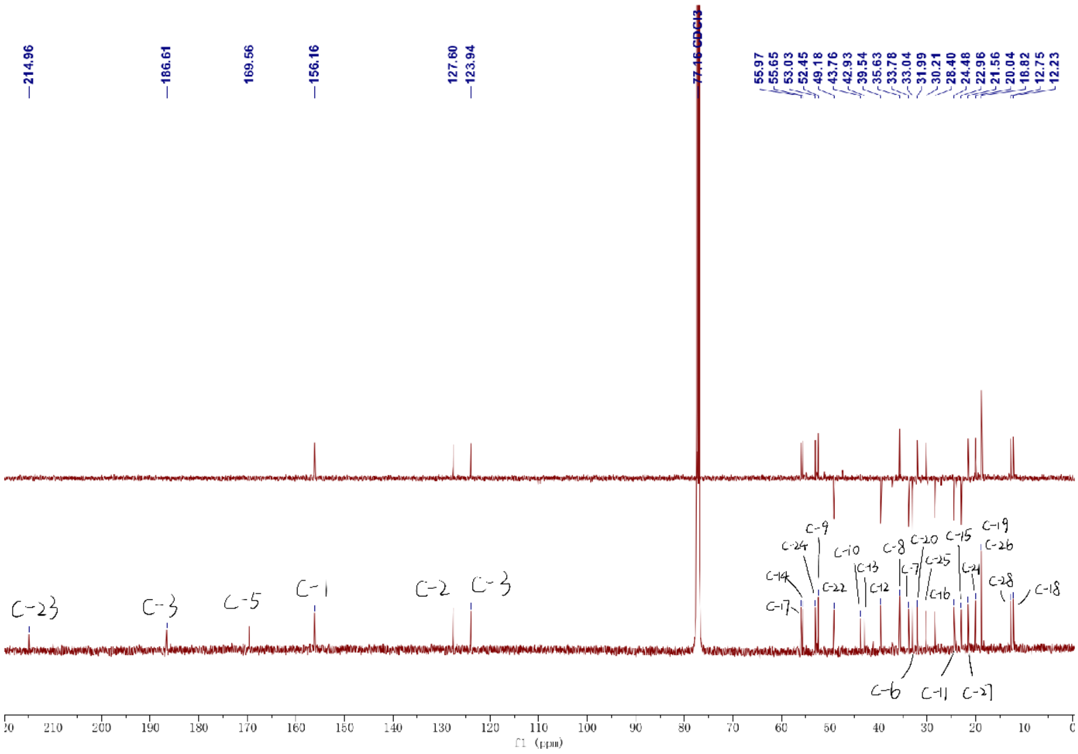

Figure S10. ^{13}C NMR spectrum (150 MHz) of compound **2** in $CDCl_3$.

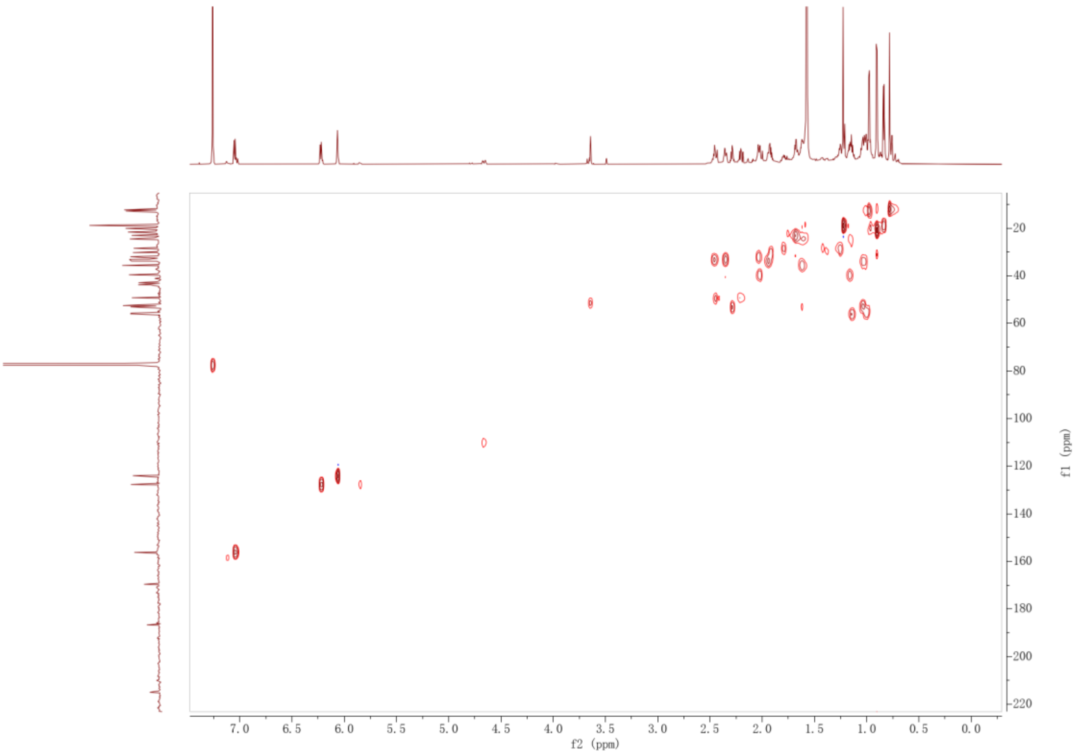

Figure S11. HSQC spectrum (800 MHz) of compound **2** in $CDCl_3$.

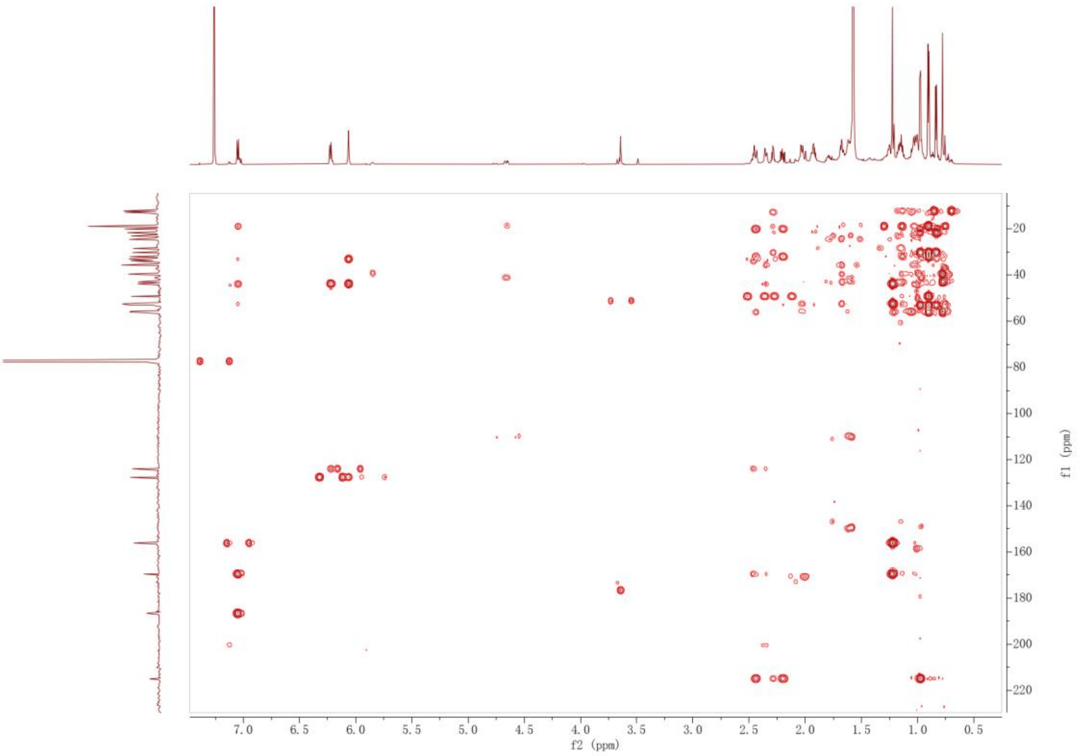

Figure S12. HMBC spectrum (800 MHz) of compound **2** in $CDCl_3$.

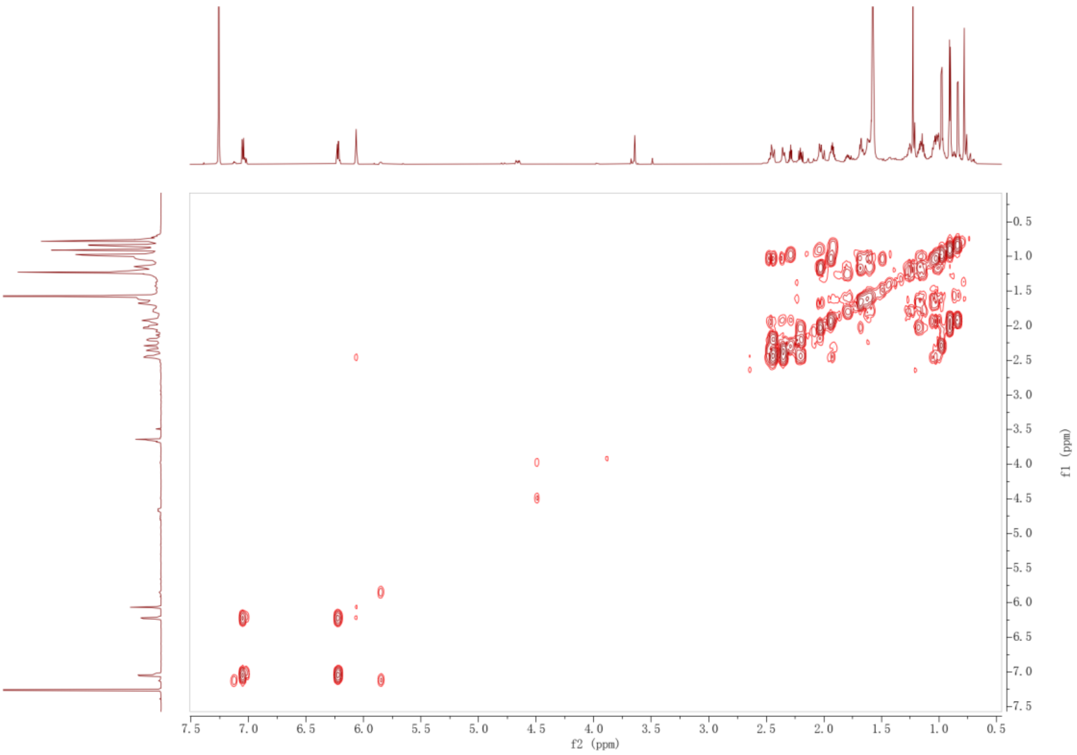

Figure S13. ^{1}H-^{1}H COSY spectrum (800 MHz) of compound **2** in $CDCl_3$.

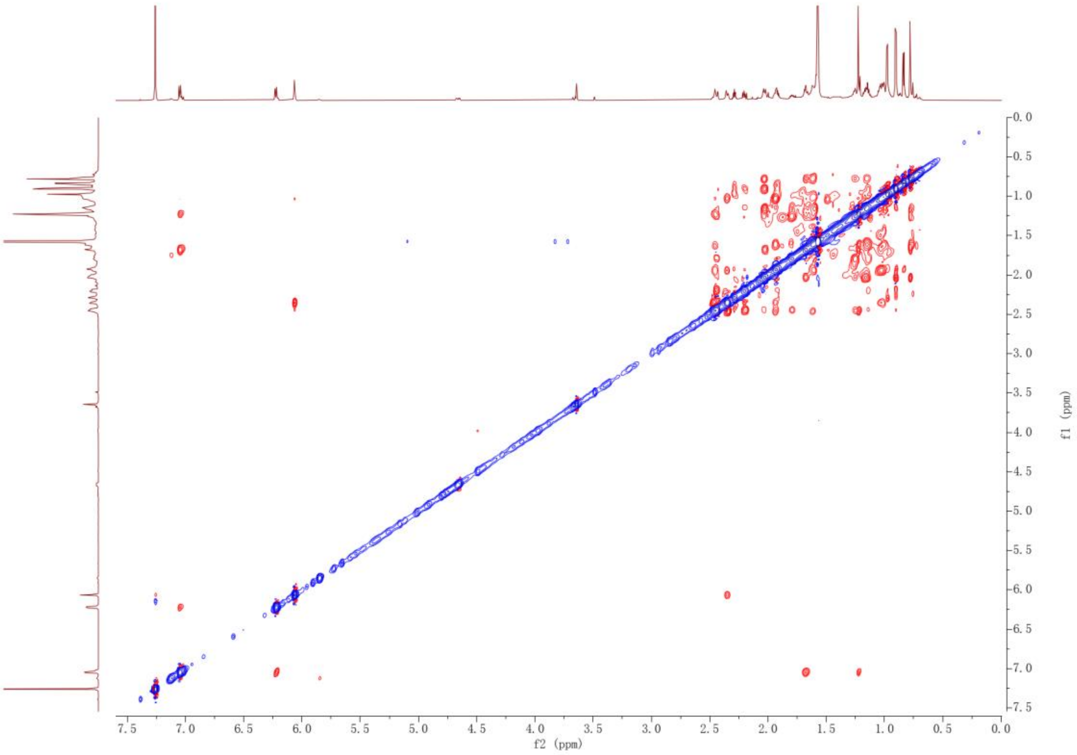

Figure S14. NOESY spectrum (800 MHz) of compound **2** in $CDCl_3$.

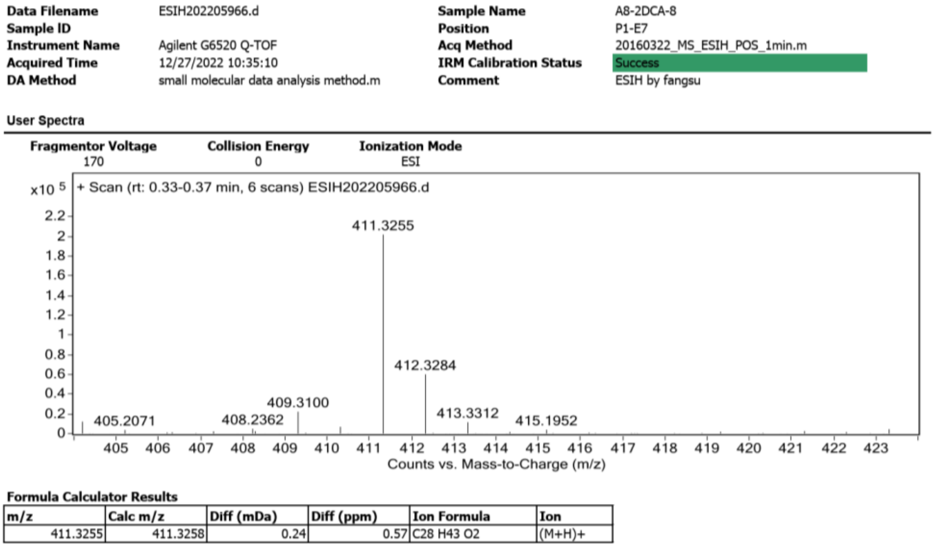

m/z	Calc m/z	Diff (mDa)	Diff (ppm)	Ion Formula	Ion
411.3255	411.3258	0.24	0.57	C28 H43 O2	(M+H)+

Figure S15. HR-ESIMS spectrum of compound **2**.

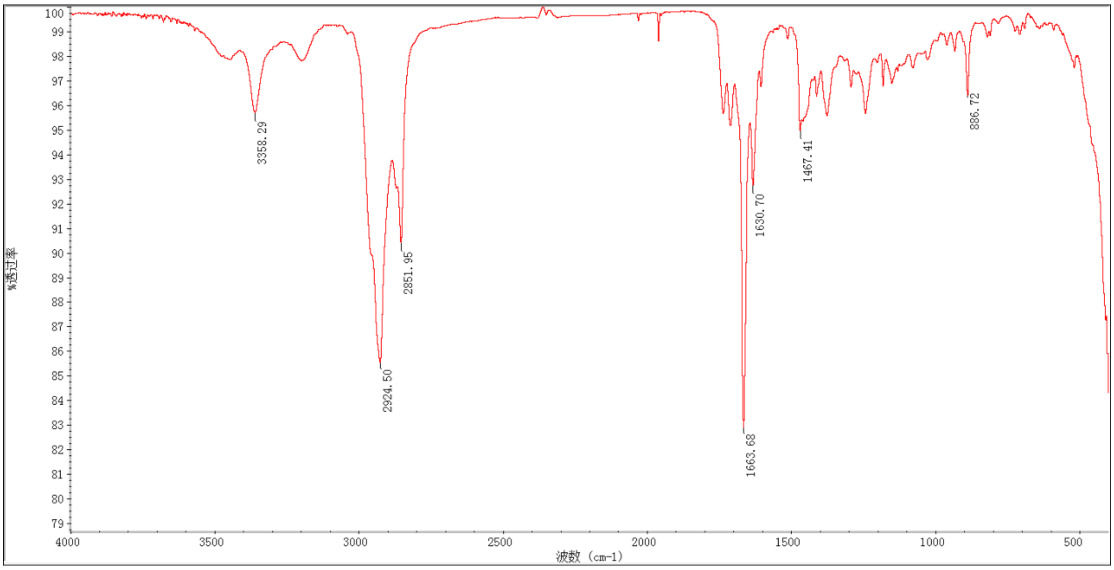

Figure S16. IR spectrum of compound **2**.

3. Spectra of compound 3

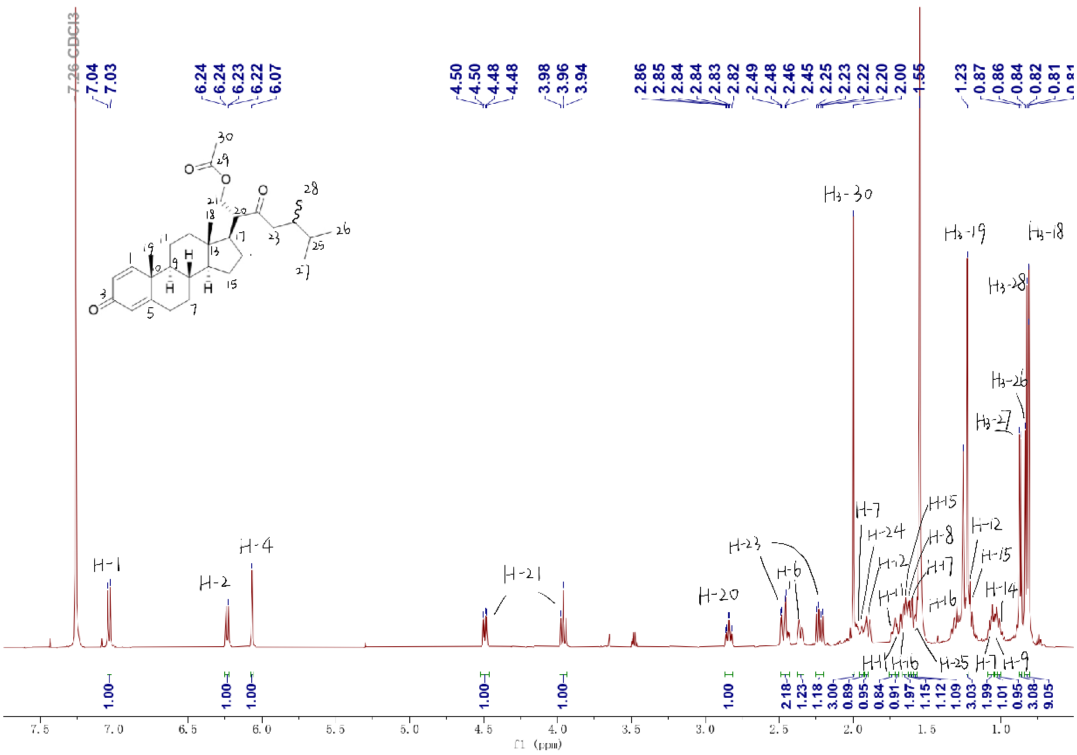

Figure S17. ^{1}H NMR spectrum (600 MHz) of compound **3** in $CDCl_3$.

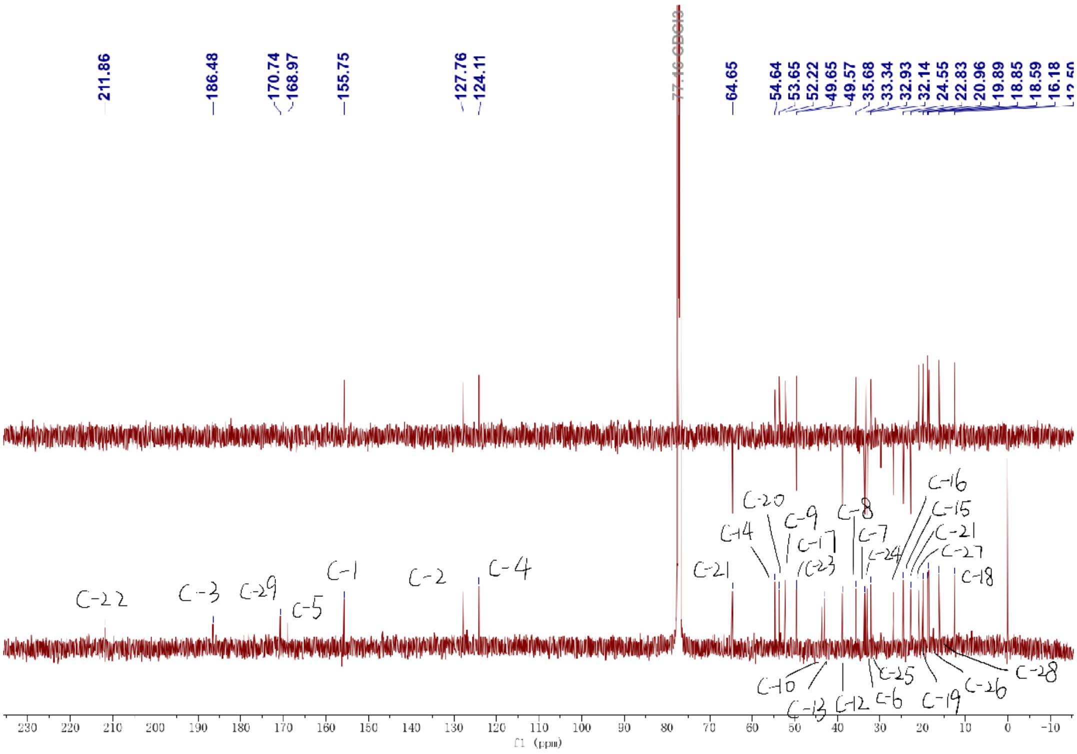

Figure S18. ^{13}C NMR spectrum (150 MHz) of compound **3** in $CDCl_3$.

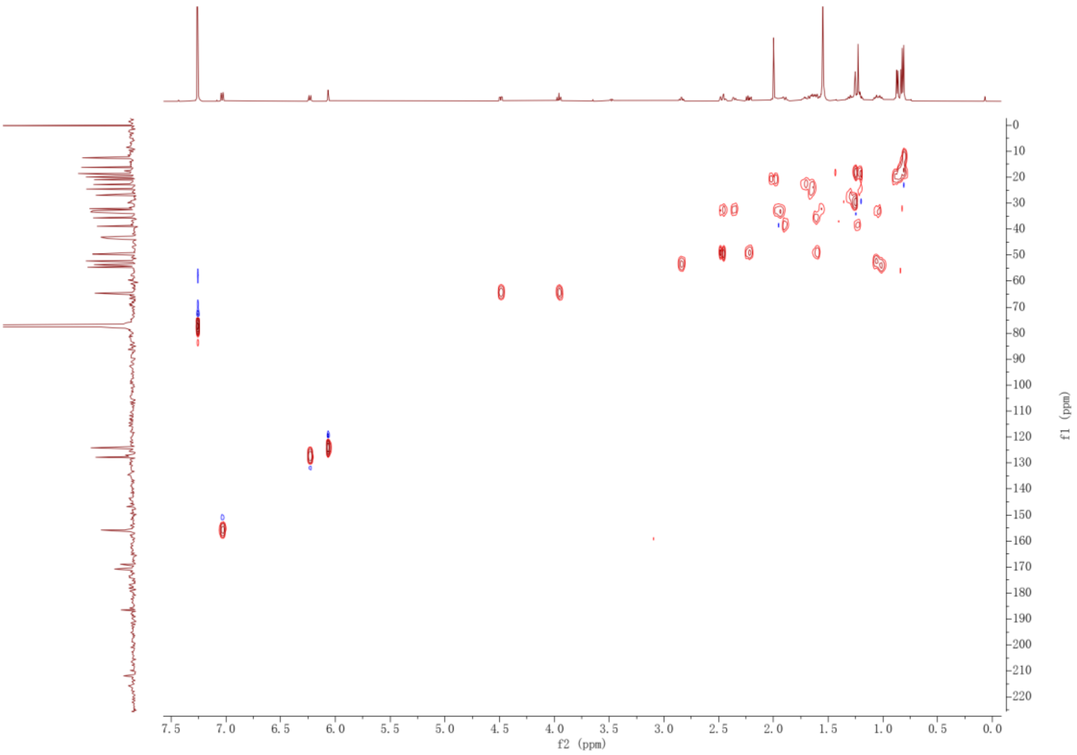

Figure S19. HSQC spectrum (600 MHz) of compound **3** in $CDCl_3$.

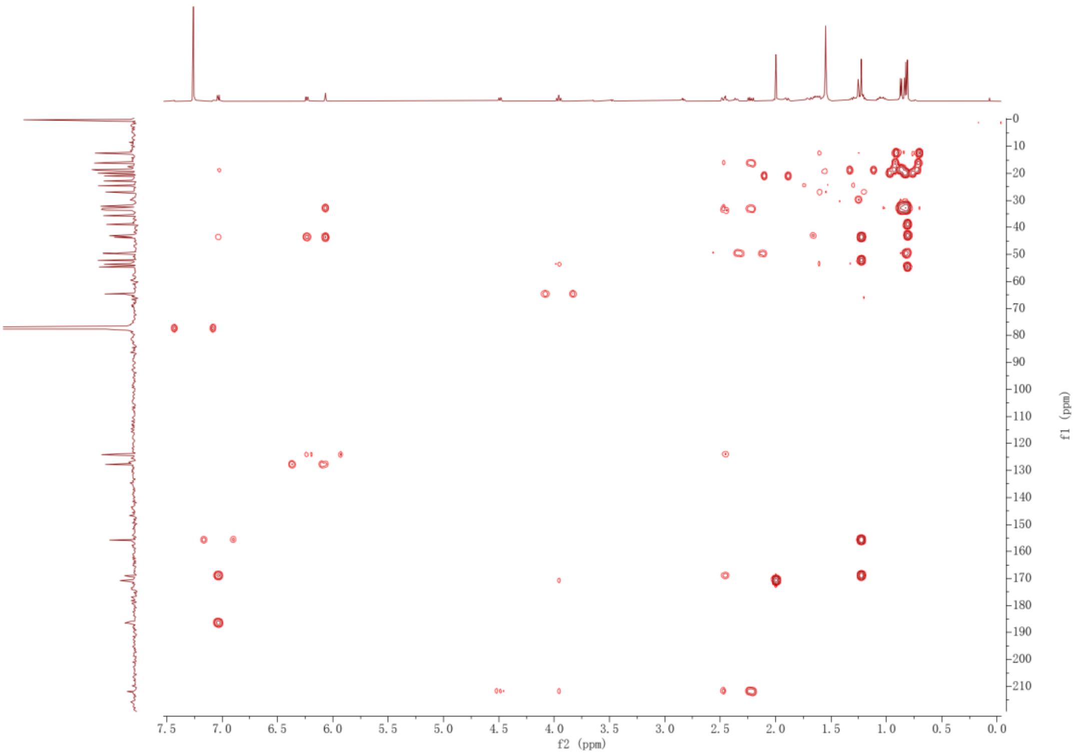

Figure S20. HMBC spectrum (600 MHz) of compound **3** in $CDCl_3$.

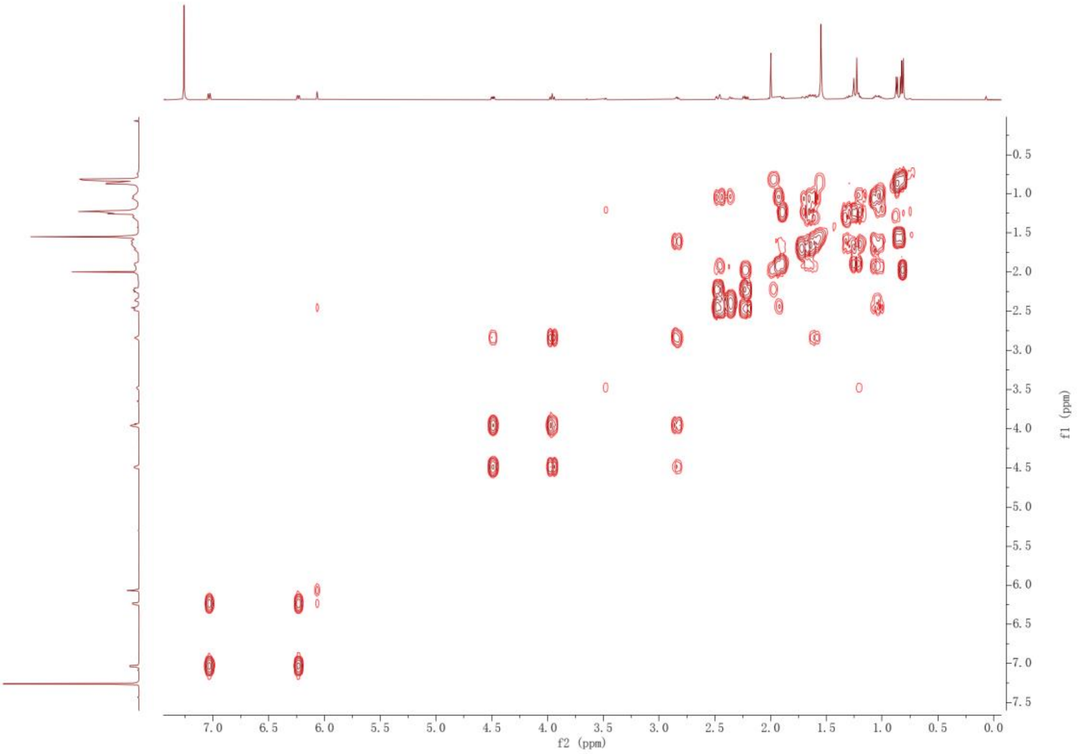

Figure S21. ^{1}H-^{1}H COSY spectrum (600 MHz) of compound **3** in $CDCl_3$.

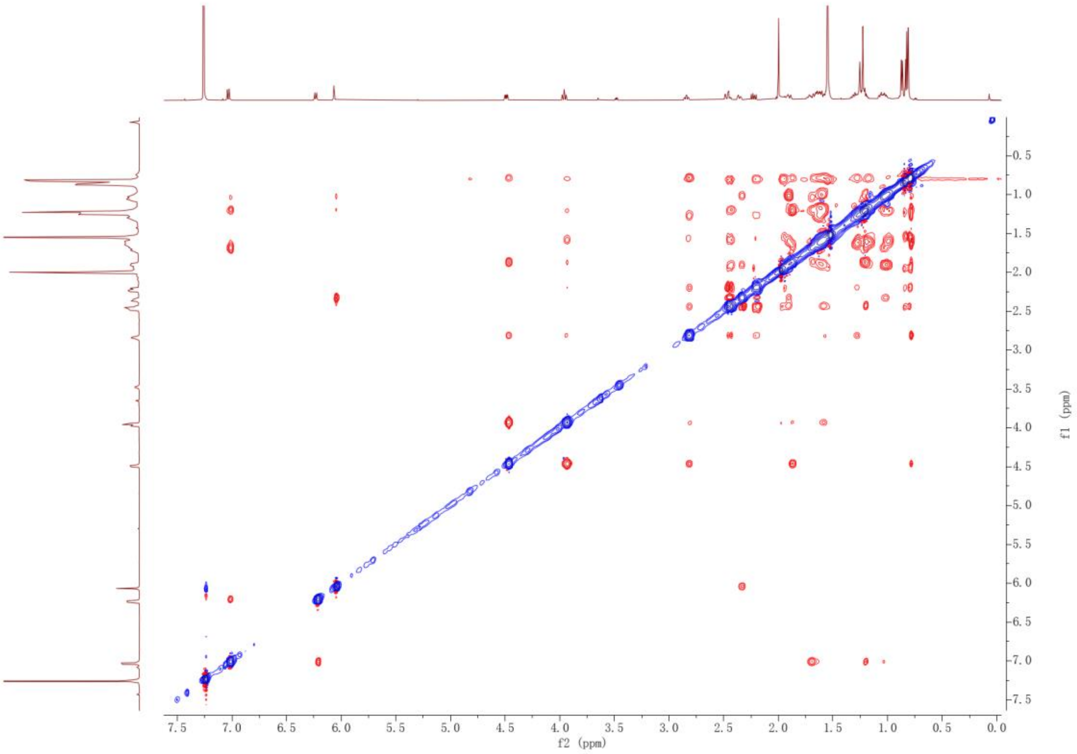

Figure S22. NOESY spectrum (600 MHz) of compound **3** in $CDCl_3$.

**Data Filename**	ESIH202205796.d	**Sample Name**	A8-AFBBBE-6
**Sample ID**		**Position**	P1-D2
**Instrument Name**	Agilent G6520 Q-TOF	**Acq Method**	20160322_MS_ESIH_POS_1min.m
**Acquired Time**	12/20/2022 15:04:07	**IRM Calibration Status**	Success
**DA Method**	small molecular data analysis method.m	**Comment**	ESIH by zhuzhenyun

User Spectra

Fragmentor Voltage	Collision Energy	Ionization Mode
170	0	ESI

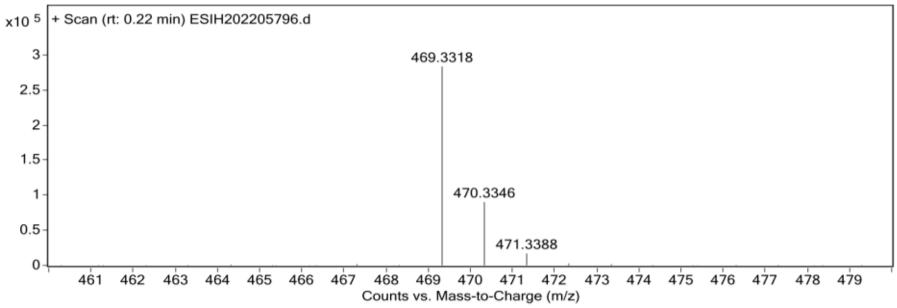

Formula Calculator Results

m/z	Calc m/z	Diff (mDa)	Diff (ppm)	Ion Formula	Ion
469.3318	469.3312	-0.56	-1.19	C30 H45 O4	(M+H)+

Figure S23. HR-ESIMS spectrum of compound **3**.

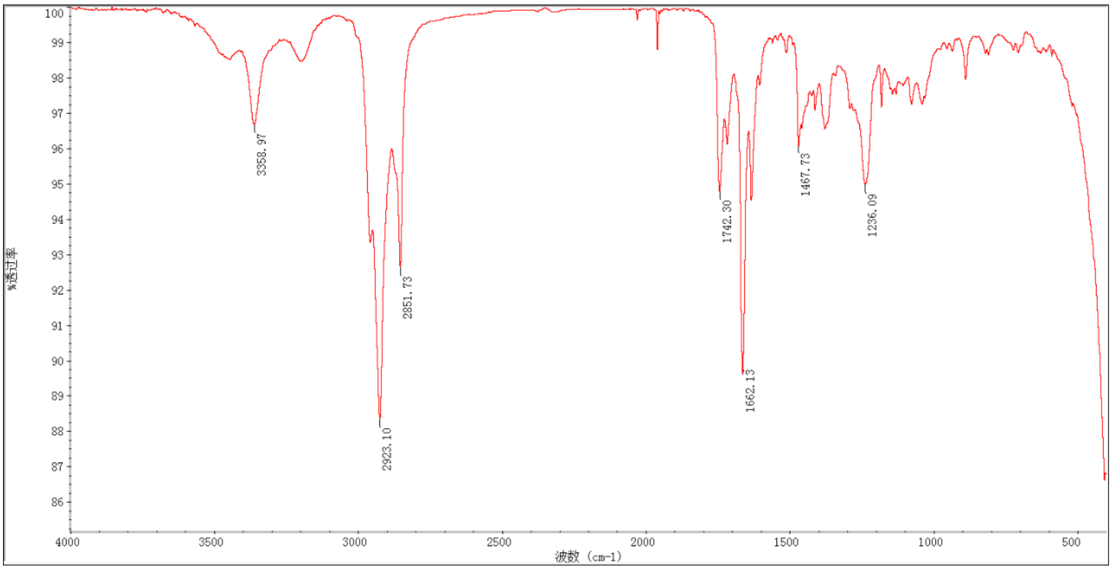

Figure S24. IR spectrum of compound **3**.

4. Spectra of compound 4

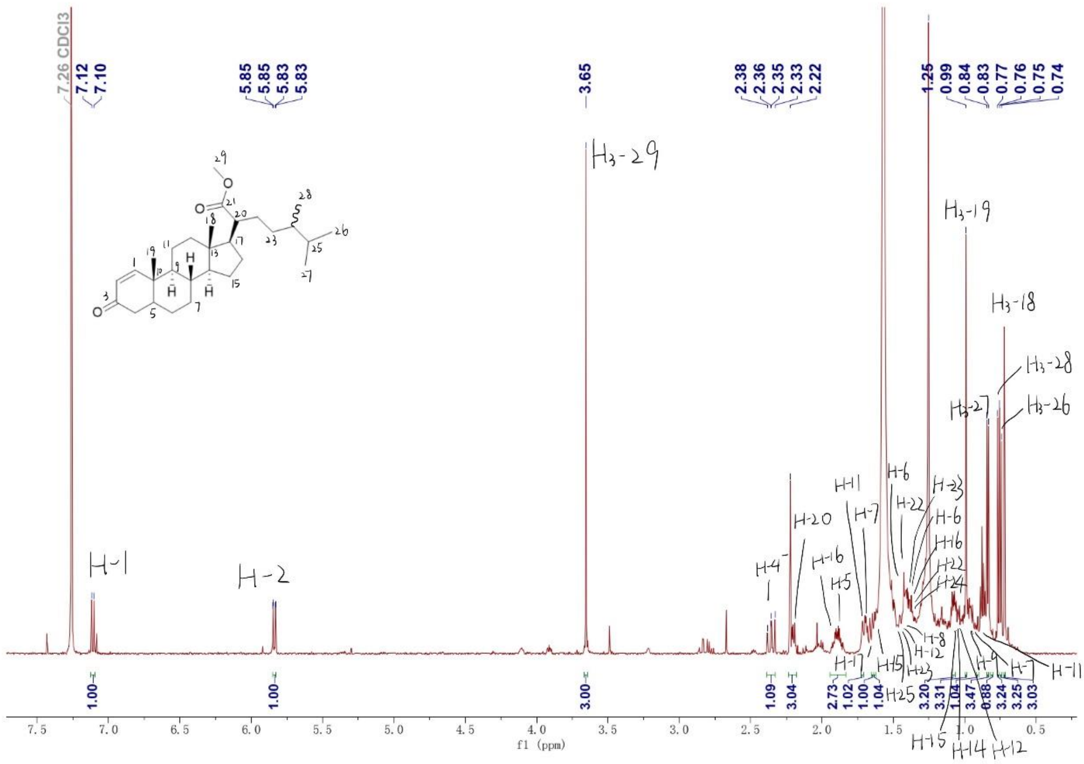

Figure S25. ^{1}H NMR spectrum (600 MHz) of compound **4** in $CDCl_3$.

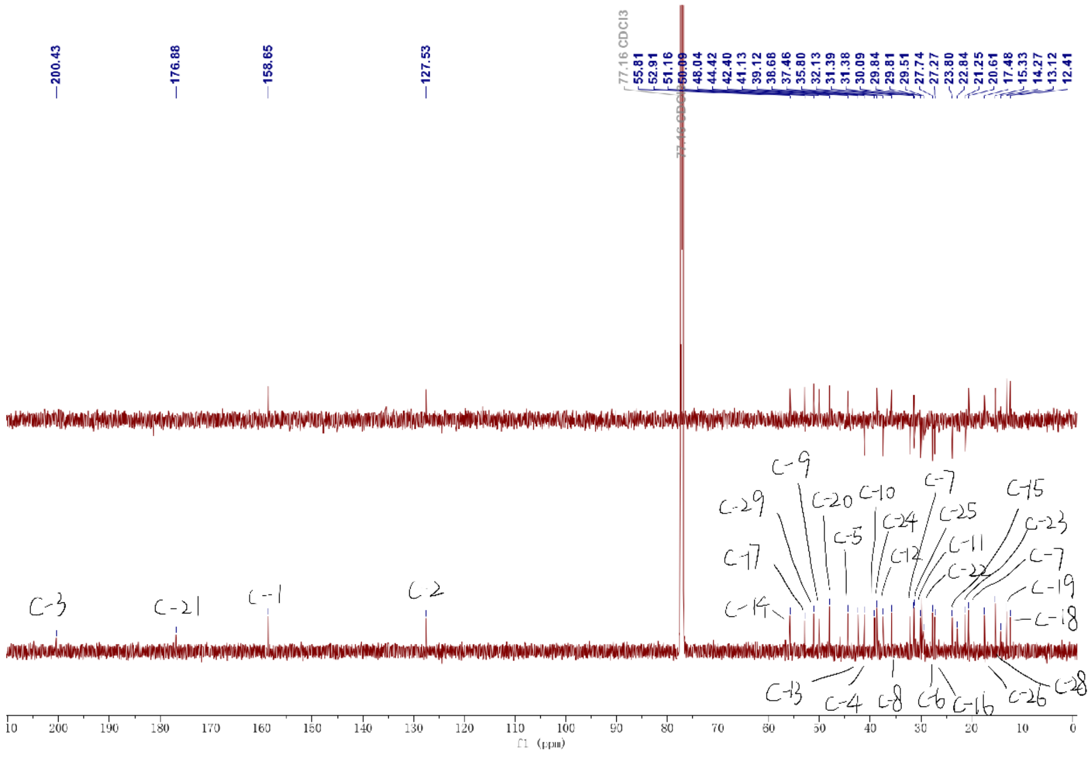

Figure S26. ^{13}C NMR spectrum (200 MHz) of compound **4** in $CDCl_3$.

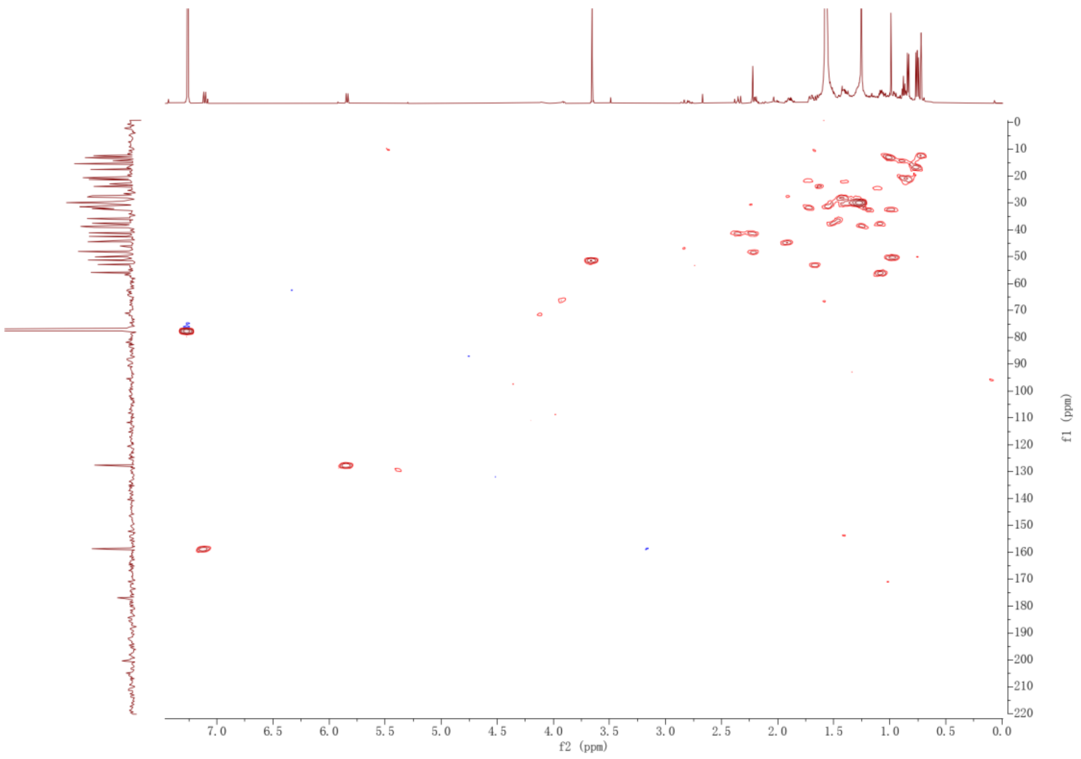

Figure S27. HSQC spectrum (600 MHz) of compound **4** in $CDCl_3$.

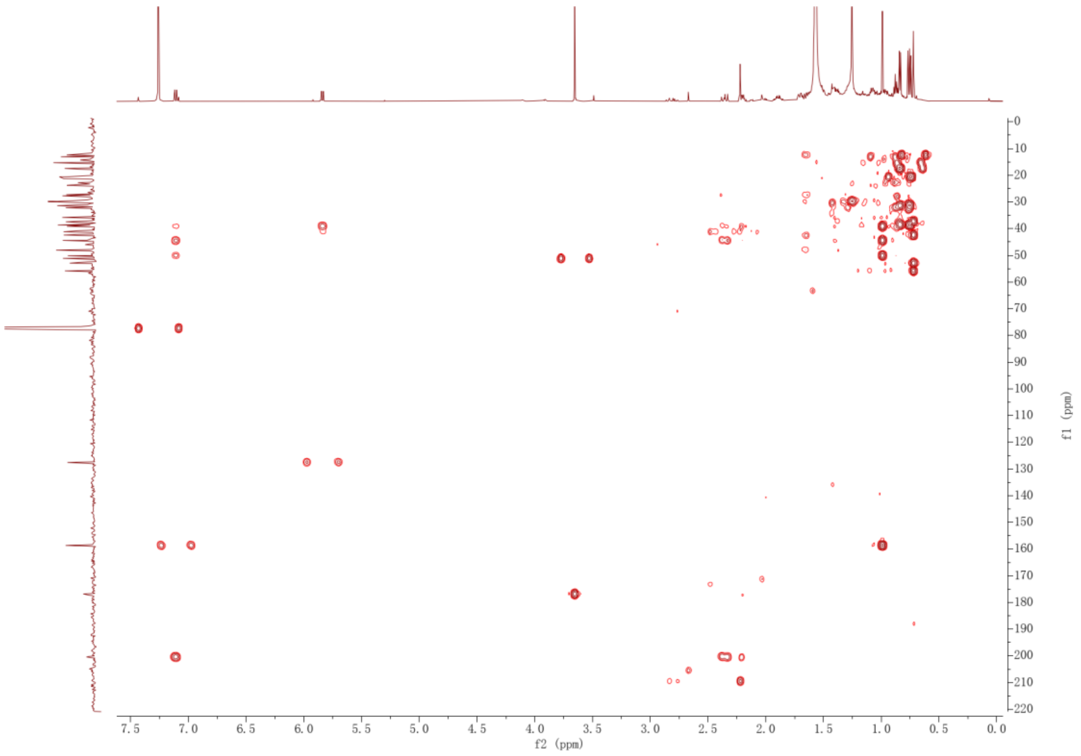

Figure S28. HMBC spectrum (600 MHz) of compound **4** in $CDCl_3$.

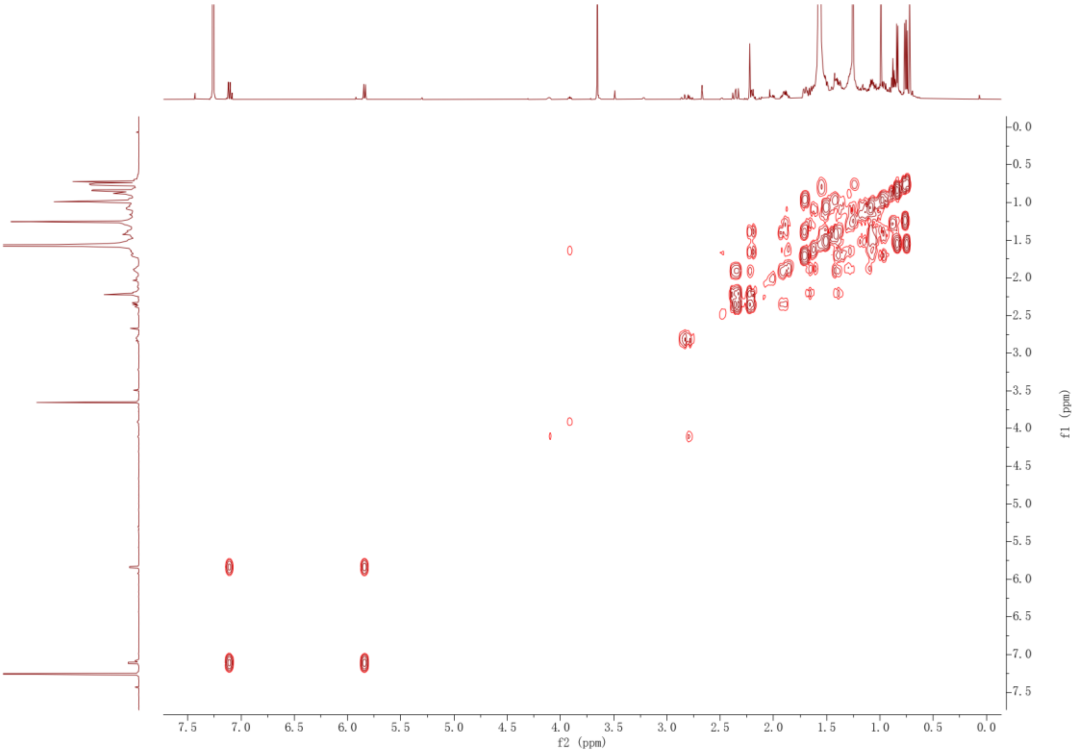

Figure S29. ^{1}H-^{1}H COSY spectrum (600 MHz) of compound **4** in $CDCl_3$.

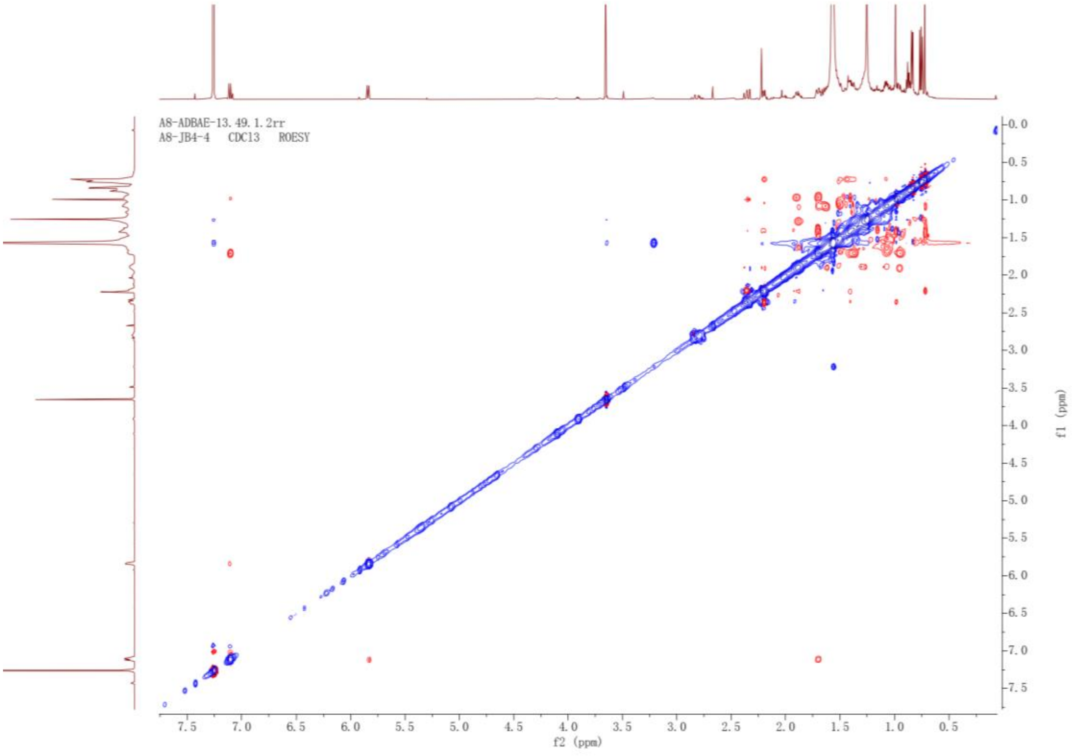

Figure S30. ROESY spectrum (600 MHz) of compound **4** in $CDCl_3$.

**Data Filename**	ESIH202302105.d	**Sample Name**	A8-A8-ADBAE-13
**Sample ID**		**Position**	P1-A1
**Instrument Name**	Agilent 6520 Q-TOF	**Acq Method**	20160322_MS_ESIH_POS_1min.m
**Acquired Time**	3/28/2023 14:46:28	**IRM Calibration Status**	Success
**DA Method**	small molecular data analysis method.m	**Comment**	ESIH by fangsu

User Spectra

Fragmentor Voltage	Collision Energy	Ionization Mode
170	0	ESI

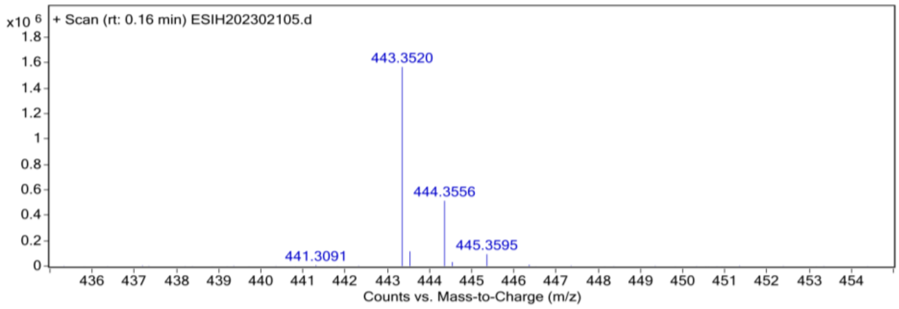

Formula Calculator Results

m/z	Calc m/z	Diff (mDa)	Diff (ppm)	Ion Formula	Ion
443.352	443.352	0	-0.01	C29 H47 O3	(M+H)+

Figure S31. HR-ESIMS spectrum of compound **4**.

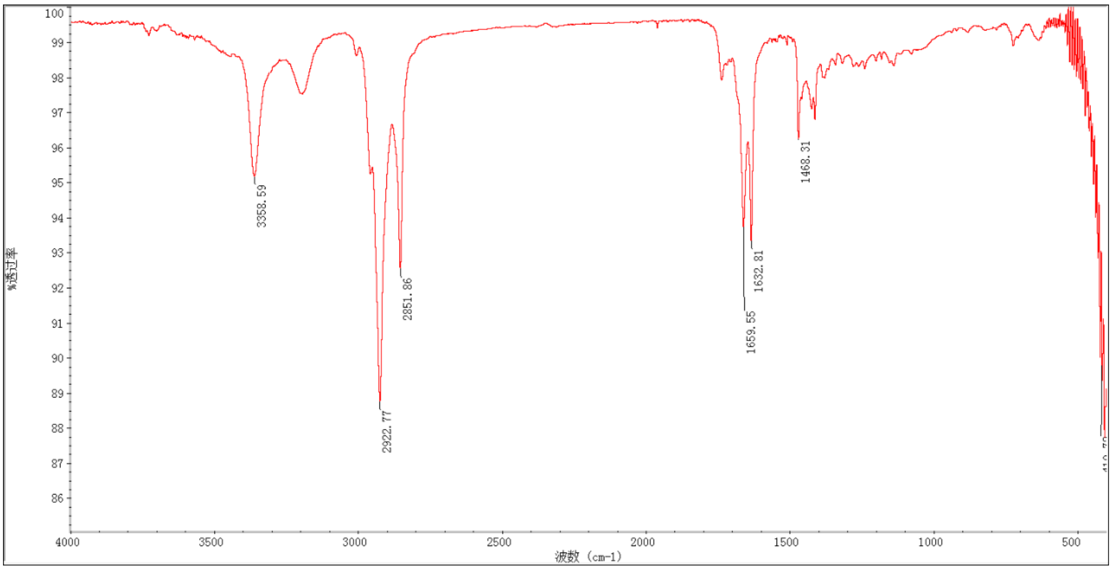

Figure S32. IR spectrum of compound **4**.

5. Spectra of compound 5

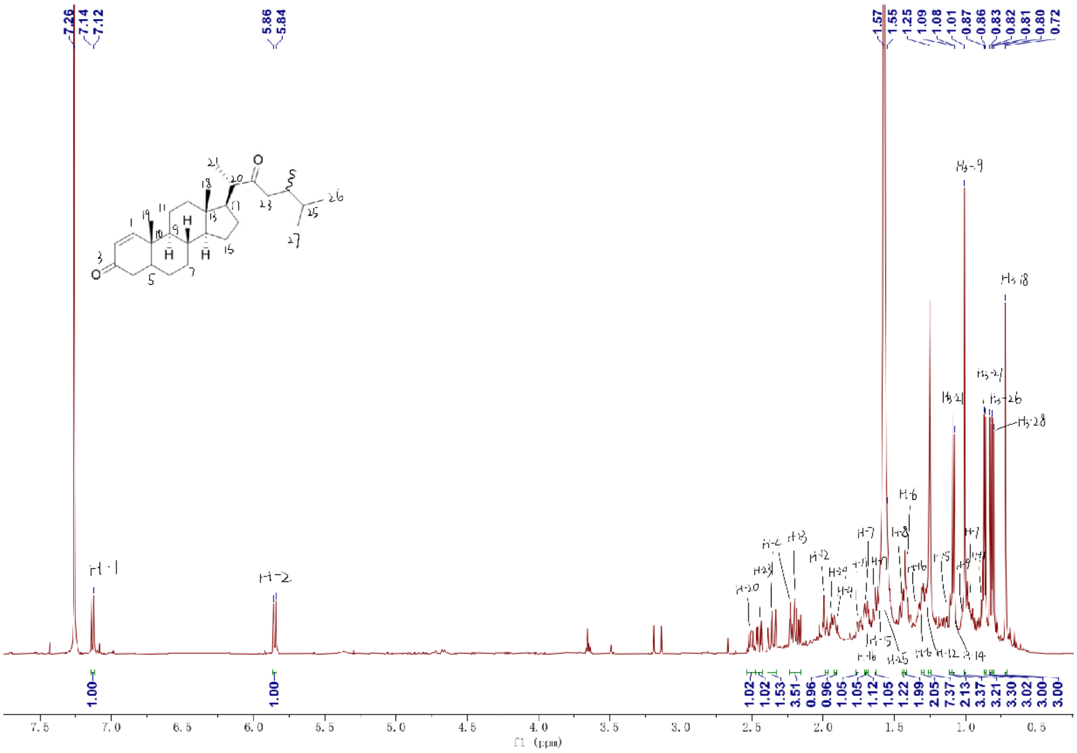

Figure S33. ^{1}H NMR spectrum (600 MHz) of compound **5** in $CDCl_3$.

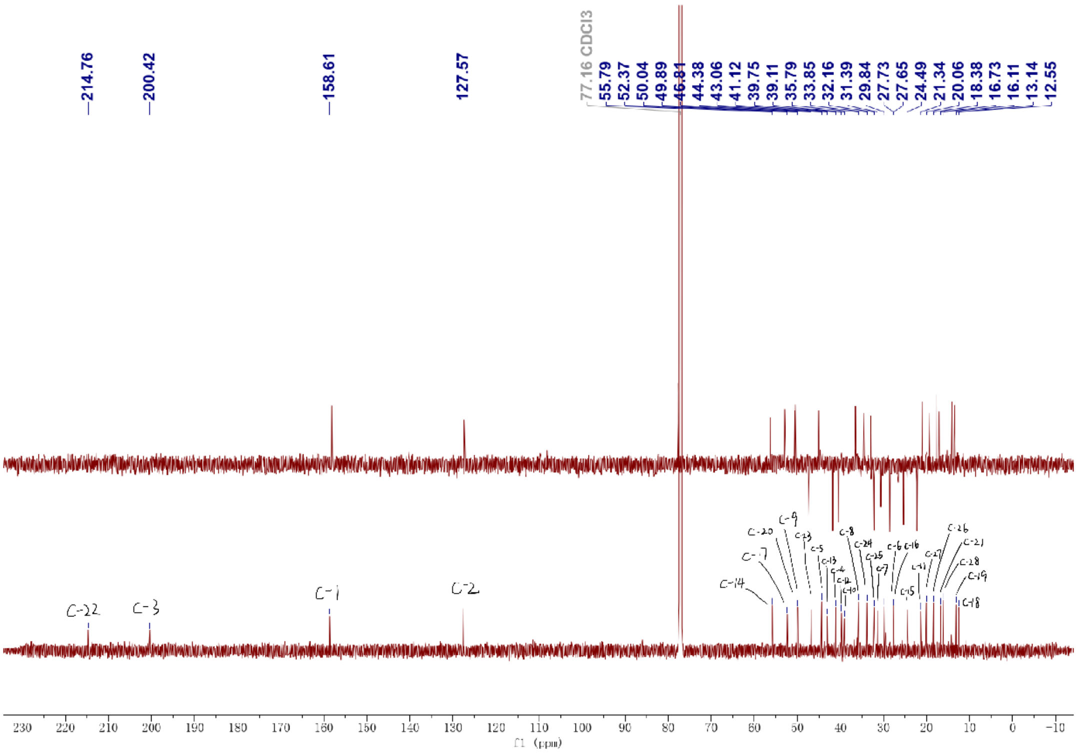

Figure S34. ^{13}C NMR spectrum (200 MHz) of compound **5** in $CDCl_3$.

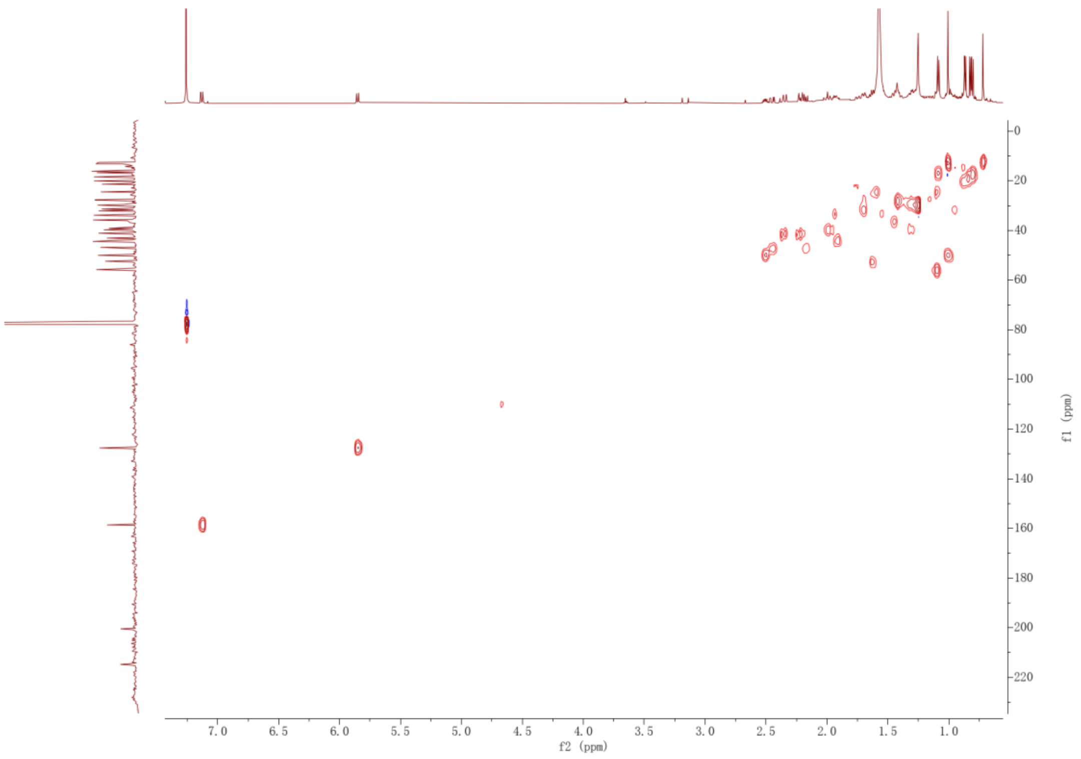

Figure S35. HSQC spectrum (600 MHz) of compound **5** in $CDCl_3$.

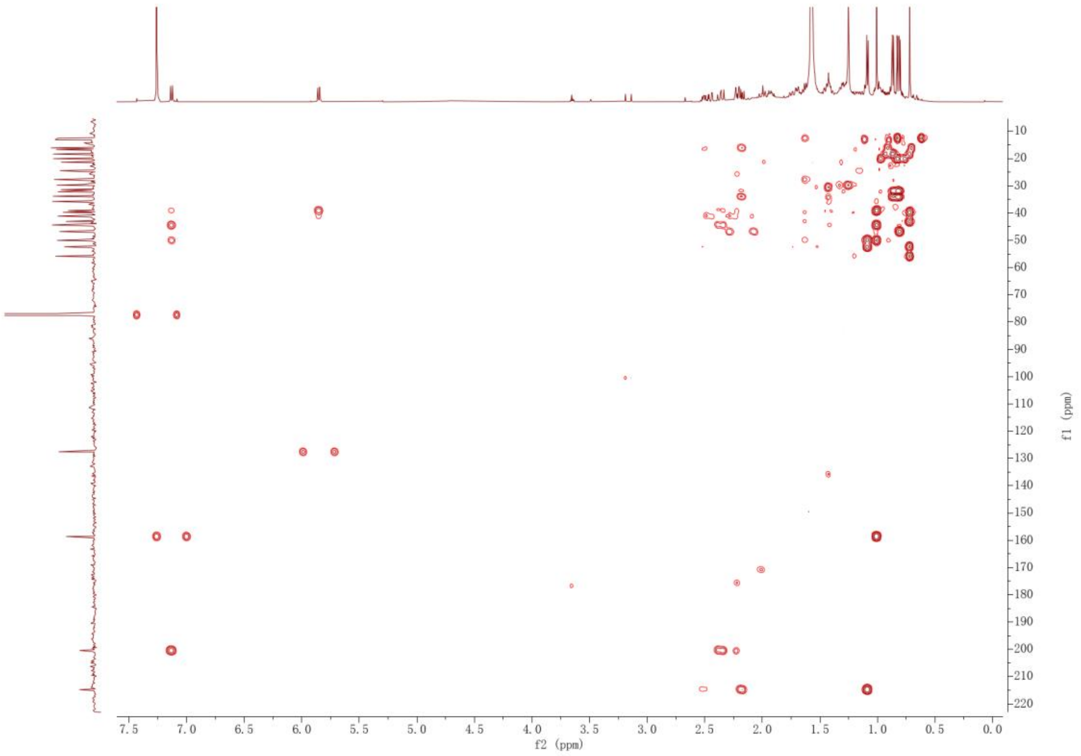

Figure S36. HMBC spectrum (600 MHz) of compound **5** in $CDCl_3$.

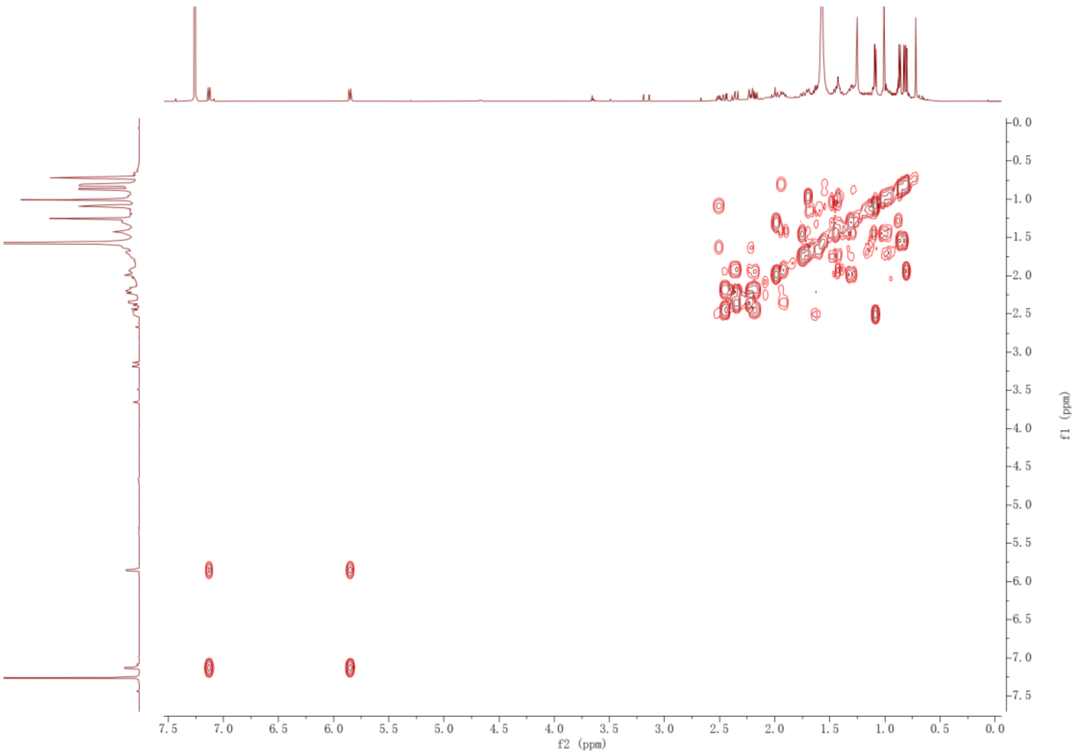

Figure S37. ^{1}H-^{1}H COSY spectrum (600 MHz) of compound **5** in $CDCl_3$.

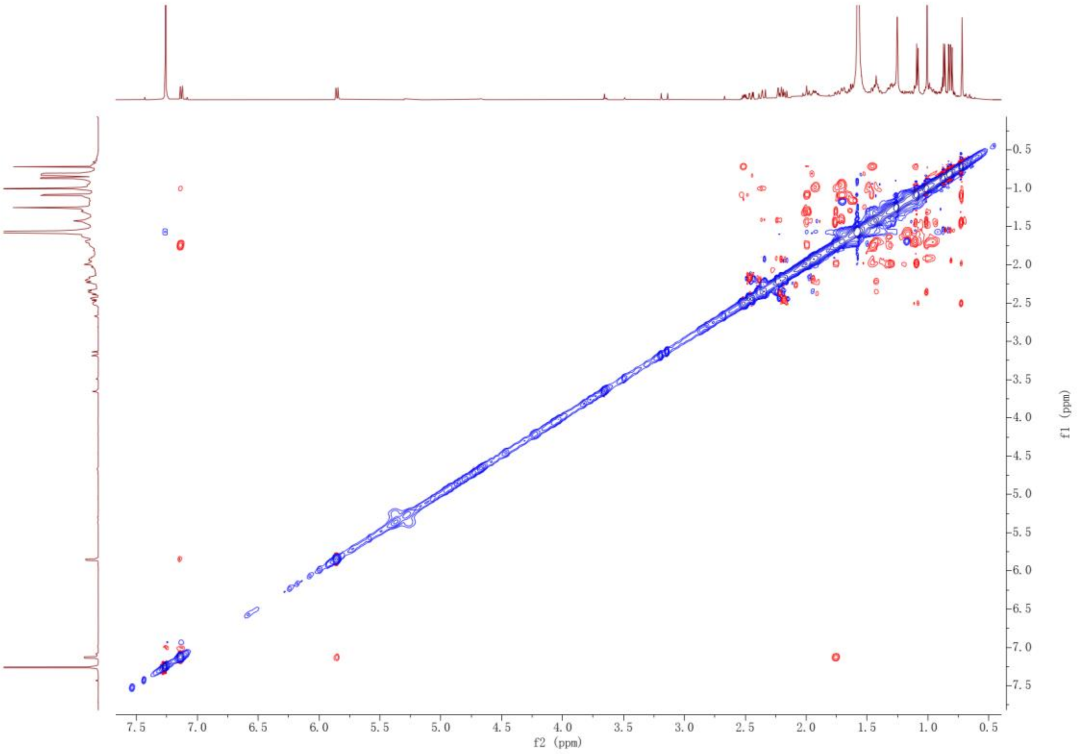

Figure S38. ROESY spectrum (600 MHz) of compound **5** in $CDCl_3$.

**Data Filename**	ESIH202203323.d	**Sample Name**	A8-A8-ADBAE12
**Sample ID**		**Position**	P1-F4
**Instrument Name**	Agilent G6520 Q-TOF	**Acq Method**	20160322_MS_ESIH_POS_1min.m
**Acquired Time**	9/14/2022 15:22:02	**IRM Calibration Status**	Success
**DA Method**	small molecular data analysis method.m	**Comment**	ESIH by fangsu

User Spectra

Fragmentor Voltage	Collision Energy	Ionization Mode
170	0	ESI

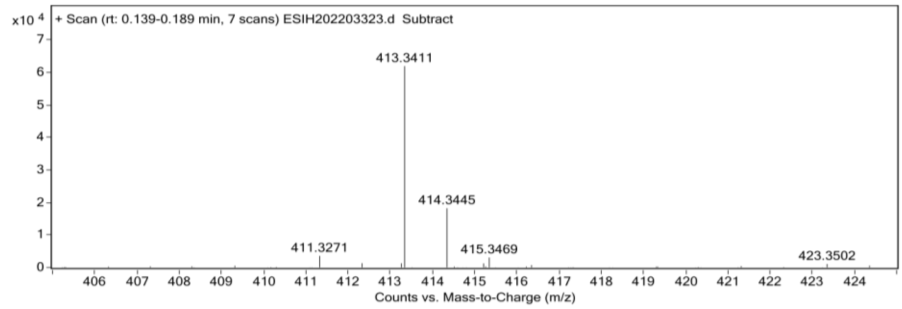

Formula Calculator Results

m/z	Calc m/z	Diff (mDa)	Diff (ppm)	Ion Formula	Ion
413.3411	413.3414	0.27	0.65	C28 H45 O2	(M+H)+

Figure S39. HR-ESIMS spectrum of compound **5**.

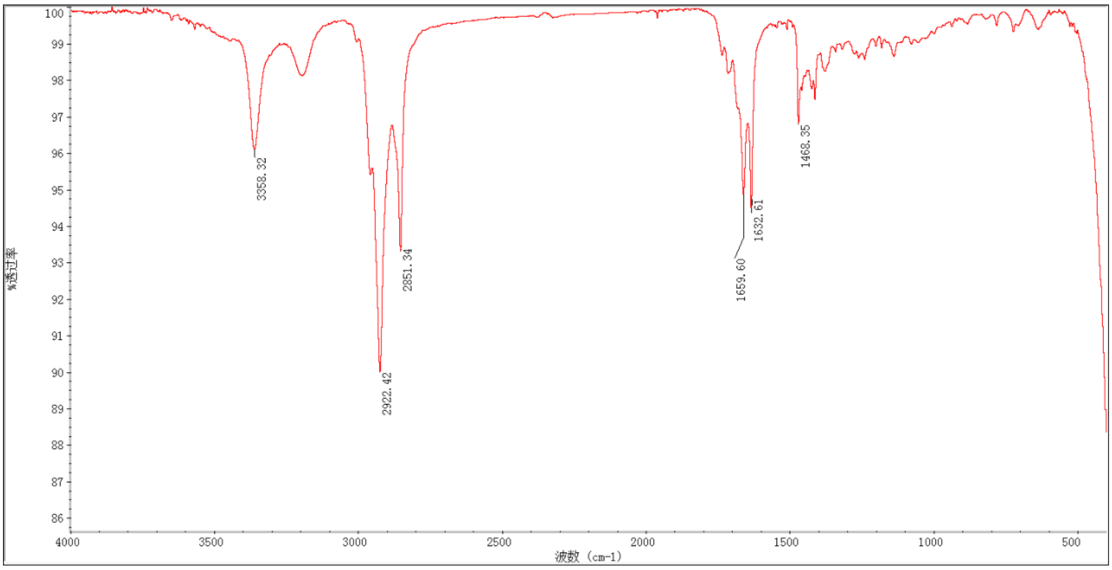

Figure S40. IR spectrum of compound **5**.

6. Spectra of compound 6

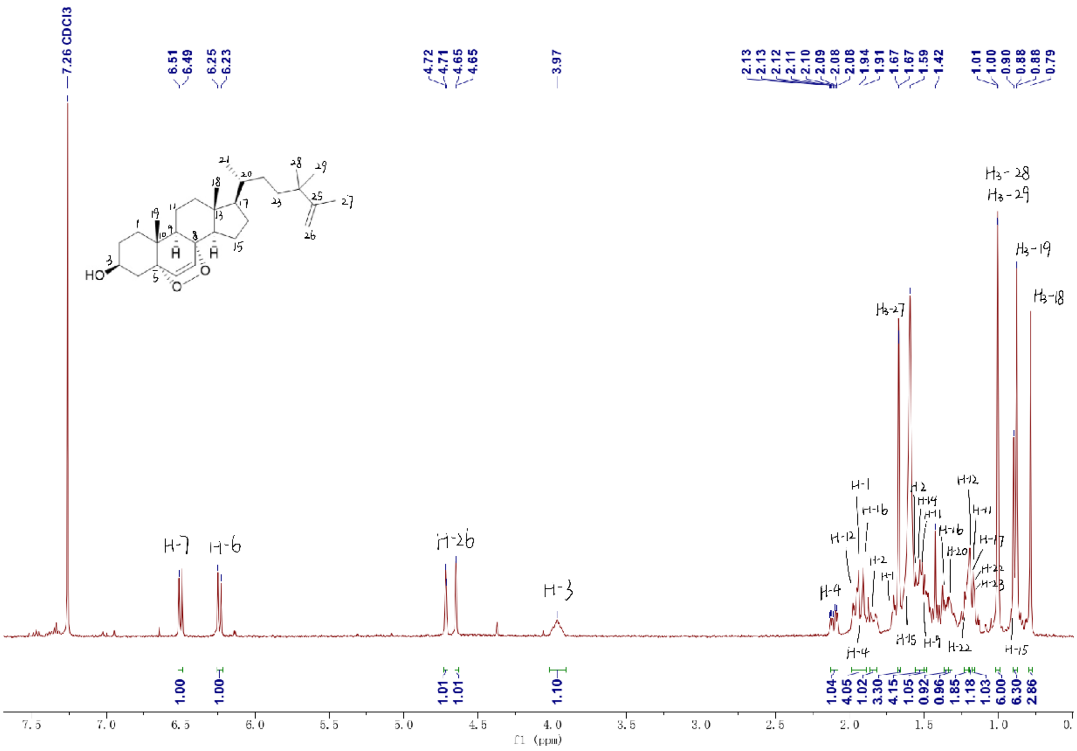

Figure S41. ^{1}H NMR spectrum (400 MHz) of compound **6** in $CDCl_3$.

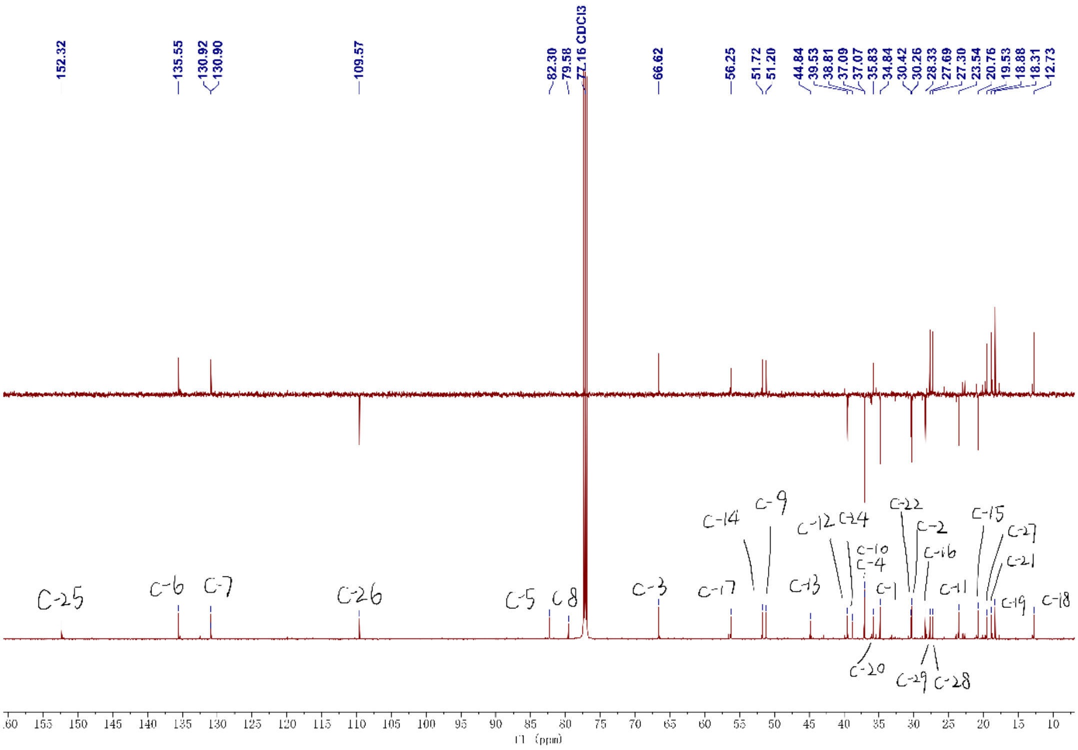

Figure S42. ^{13}C NMR spectrum (150 MHz) of compound **6** in $CDCl_3$.

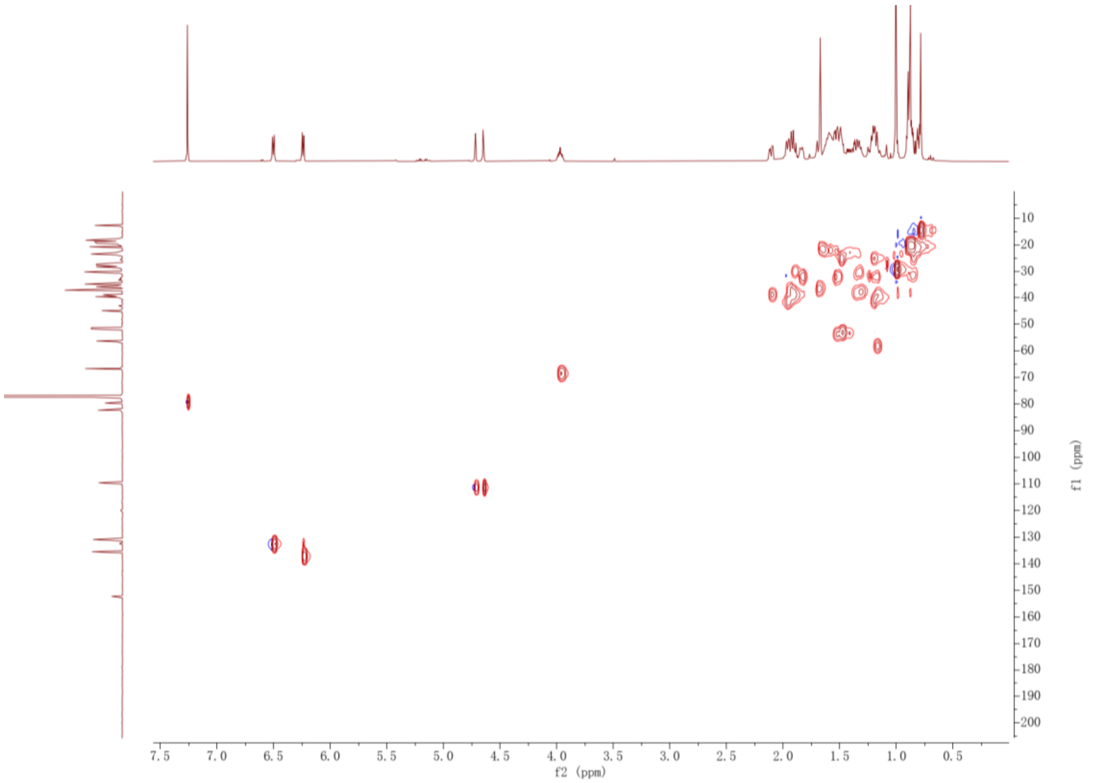

Figure S43. HSQC spectrum (600 MHz) of compound **6** in $CDCl_3$.

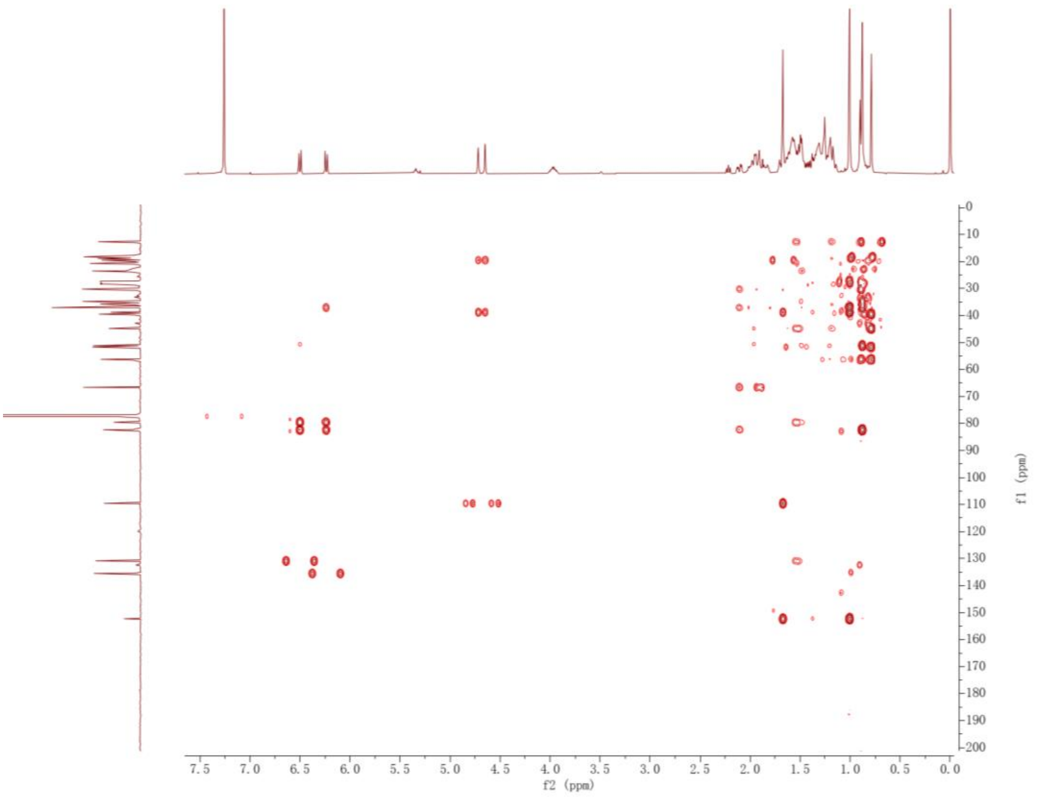

Figure S44. HMBC spectrum (600 MHz) of compound **6** in $CDCl_3$.

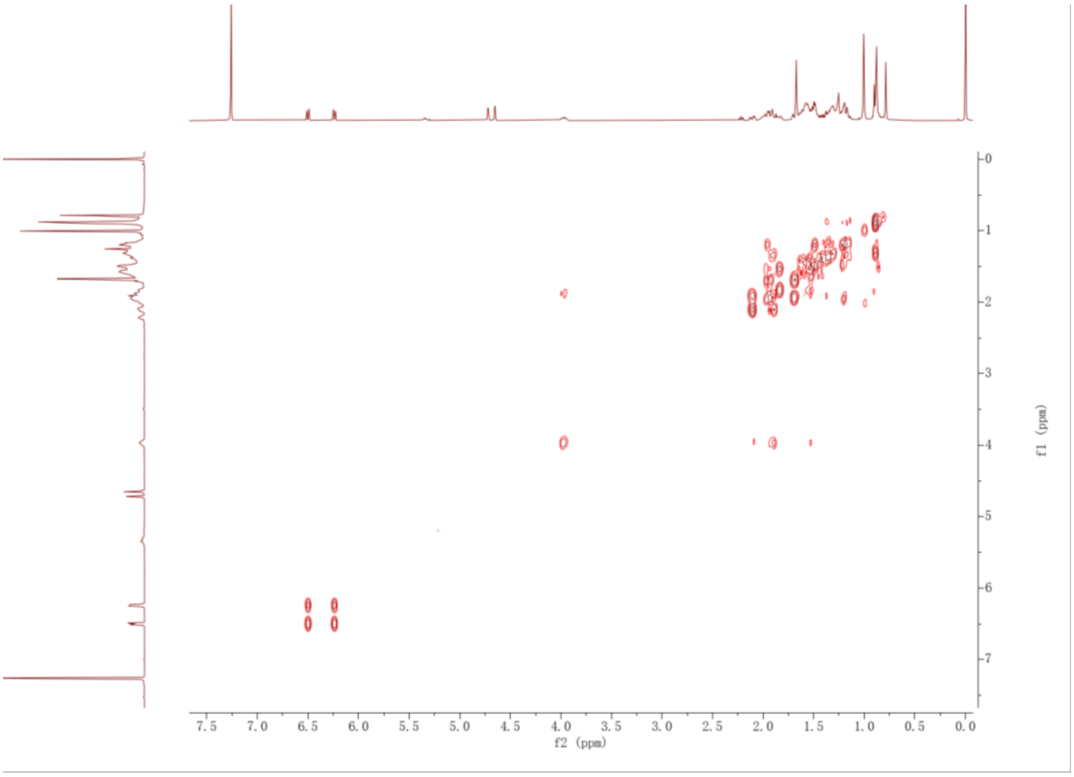

Figure S45. ^1H-^1H COSY spectrum (600 MHz) of compound **6** in $CDCl_3$.

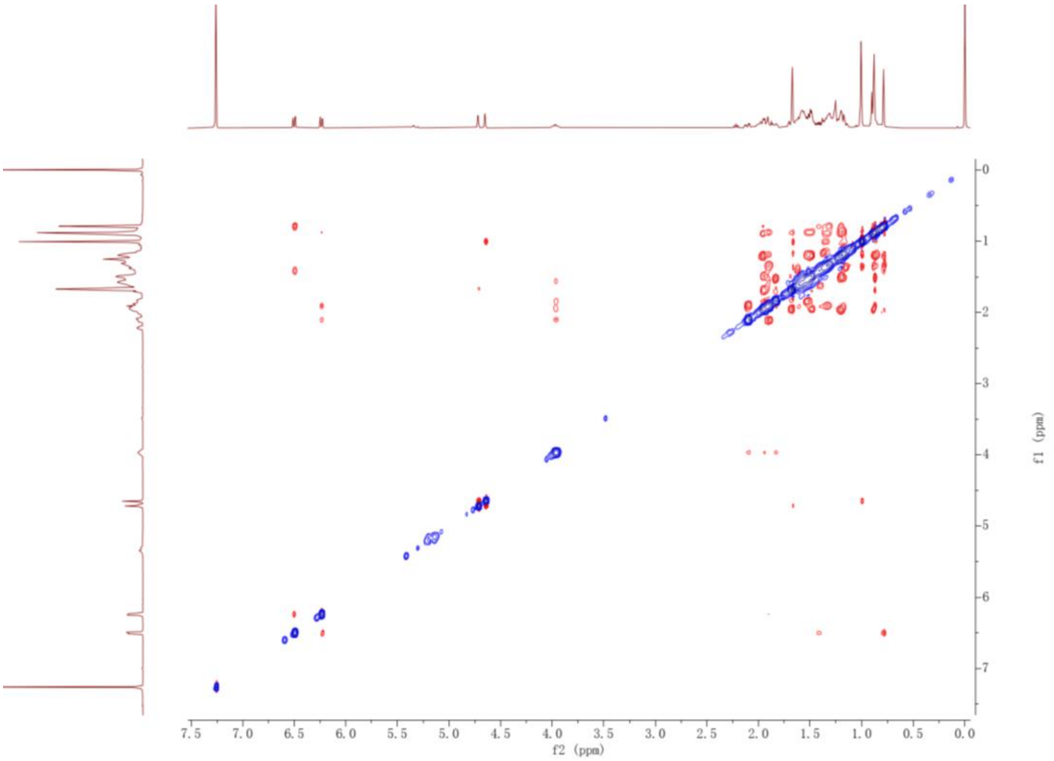

Figure S46. NOESY spectrum (600 MHz) of compound **6** in $CDCl_3$.

EI202300247_A8-2CBgCD-7 -c1#17 RT: 3.30
T: + c EI Full ms [49.50-800.50]
m/z= 48-803

m/z	Intensity	Relative	Theo. Mass	Delta (mmu)	RDB equiv.	Composition
69.0692	2526253.0	14.16	69.0699	-0.67	1.5	C_5H_9
79.0176	1227372.0	6.88	79.0178	-0.23	4.5	$C_5H_3O_1$
93.0697	1596930.0	8.95	93.0699	-0.15	3.5	C_7H_9
95.0865	1721079.0	9.65	95.0855	0.97	2.5	C_7H_{11}
119.0858	1105517.0	6.20	119.0855	0.27	4.5	C_9H_{11}
121.1009	849310.0	4.76	121.1012	-0.23	3.5	C_9H_{13}
123.1161	662506.0	3.71	123.1168	-0.69	2.5	C_9H_{15}
131.0844	863563.0	4.84	131.0855	-1.09	5.5	$C_{10}H_{11}$
133.1005	884241.0	4.96	133.1012	-0.70	4.5	$C_{10}H_{13}$
137.1317	646525.0	3.62	137.1325	-0.77	2.5	$C_{10}H_{17}$
143.0848	1464170.0	8.21	143.0855	-0.71	6.5	$C_{11}H_{11}$
145.1009	1483471.0	8.32	145.1012	-0.29	5.5	$C_{11}H_{13}$
152.0828	1155889.0	6.48	152.0832	-0.35	4.0	$C_9H_{12}O_2$
157.1010	1241274.0	6.96	157.1012	-0.20	6.5	$C_{12}H_{13}$
158.1074	1285518.0	7.21	158.1090	-1.55	6.0	$C_{12}H_{14}$
159.1150	1343098.0	7.53	159.1168	-1.79	5.5	$C_{12}H_{15}$
161.0947	745542.0	4.18	161.0961	-1.39	5.5	$C_{11}H_{13}O_1$
171.1161	1103951.0	6.19	171.1168	-0.71	6.5	$C_{13}H_{15}$
183.1162	778880.0	4.37	183.1168	-0.63	7.5	$C_{14}H_{15}$
185.1315	889154.0	4.99	185.1325	-1.01	6.5	$C_{14}H_{17}$
197.1324	1271589.0	7.13	197.1325	-0.06	7.5	$C_{15}H_{17}$
199.1478	937934.0	5.26	199.1481	-0.32	6.5	$C_{15}H_{19}$
209.1330	627898.0	3.52	209.1325	0.51	8.5	$C_{16}H_{17}$
211.1466	1328899.0	7.45	211.1481	-1.50	7.5	$C_{16}H_{19}$
249.1640	669200.0	3.75	249.1638	0.25	9.5	$C_{19}H_{21}$
251.1779	1080331.0	6.06	251.1794	-1.50	8.5	$C_{19}H_{23}$
253.1954	798748.0	4.48	253.1951	0.36	7.5	$C_{19}H_{25}$
267.1750	725700.0	4.07	267.1743	0.68	8.5	$C_{19}H_{23}O_1$
271.2060	683373.0	3.83	271.2056	0.40	6.5	$C_{19}H_{27}O_1$
293.1911	626252.0	3.51	293.1900	1.13	9.5	$C_{21}H_{25}O_1$
341.2836	751534.0	4.21	341.2839	-0.32	6.5	$C_{24}H_{37}O_1$
351.3039	3330212.0	18.67	351.3046	-0.69	7.5	$C_{26}H_{39}$
377.3198	7690815.0	43.12	377.3203	-0.48	8.5	$C_{28}H_{41}$
390.3278	639938.0	3.59	390.3281	-0.29	9.0	$C_{29}H_{42}$
391.3008	912396.0	5.12	391.2995	1.21	9.5	$C_{28}H_{39}O_1$
392.3418	643150.0	3.61	392.3438	-1.96	8.0	$C_{29}H_{44}$
395.3318	706885.0	3.96	395.3308	0.99	7.5	$C_{28}H_{43}O_1$
406.3231	953240.0	5.34	406.3230	0.06	9.0	$C_{29}H_{42}O_1$
410.3547	17835264.0	100.00	410.3543	0.36	7.0	$C_{29}H_{46}O_1$
424.3325	1101792.0	6.18	424.3336	-1.11	8.0	$C_{29}H_{44}O_2$

Figure S47. HR-EIMS spectrum of compound **6**.

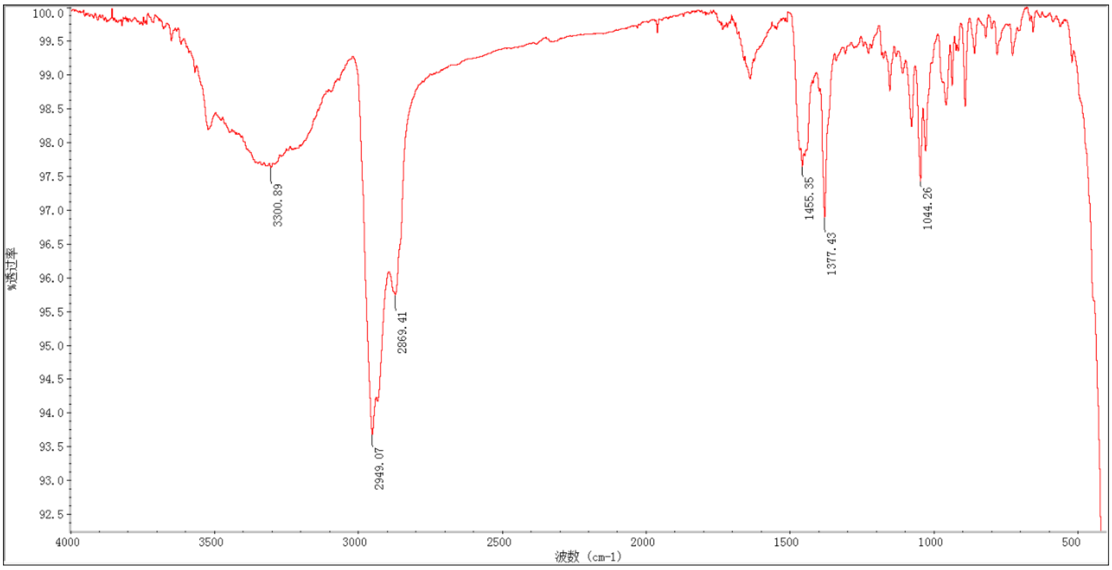

Figure S48. IR spectrum of compound **6**.
